# Logical word learning: The case of kinship

**DOI:** 10.3758/s13423-021-02017-5

**Published:** 2021-12-16

**Authors:** Francis Mollica, Steven T. Piantadosi

**Affiliations:** 1 University of Edinburgh, Edinburgh, Scotland; 2 University of California, Berkeley, CA USA

**Keywords:** Word-learning, Conceptual development, Bayesian modeling

## Abstract

We examine the conceptual development of kinship through the lens of program induction. We present a computational model for the acquisition of kinship term concepts, resulting in the first computational model of kinship learning that is closely tied to developmental phenomena. We demonstrate that our model can learn several kinship systems of varying complexity using cross-linguistic data from English, Pukapuka, Turkish, and Yanomamö. More importantly, the behavioral patterns observed in children learning kinship terms, under-extension and over-generalization, fall out naturally from our learning model. We then conducted interviews to simulate realistic learning environments and demonstrate that the characteristic-to-defining shift is a consequence of our learning model in naturalistic contexts containing abstract and concrete features. We use model simulations to understand the influence of logical simplicity and children’s learning environment on the order of acquisition of kinship terms, providing novel predictions for the learning trajectories of these words. We conclude with a discussion of how this model framework generalizes beyond kinship terms, as well as a discussion of its limitations.

In order to acquire a language, learners have to map words to objects and situations in the world. From these mappings, they must then learn the underlying concept of the word that will generalize to new objects and situations. The mappings between words and concepts, acquired over a lifetime, will constitute the majority of information a language user stores about linguistic representations (Mollica & Piantadosi, 2019). While there is vast literature on how children might solve the problem of mapping words to the world (e.g., Carey & Bartlett, 1978; Smith & Yu, 2008; Frank, Goodman, & Tenenbaum, 2009; Medina, Snedeker, Trueswell, & Gleitman, 2011; Siskind, 1996), we know less about how children use these mappings to inform their concepts in order to generalize words to new contexts. Research on children’s early word generalization has focused on uncovering biases in children’s generalizations (e.g., taxonomic constraints, Markman, 1991) and explaining the mechanism and types of input children need to overcome these biases (e.g., Gentner & Namy, 1999; Graham, Namy, Gentner, & Meagher, 2010); however, research has yet to precisely predict children’s behavior across the developmental trajectory. We propose a theoretical model from two first principles—simplicity and strong sampling, to scale up our understanding of how children’s word meanings should change as they observe more data. In the process, we demonstrate that several seemingly unrelated patterns in children’s early word use can be explained by the process of induction in naturalistic learning contexts.

Understanding how children’s conceptual knowledge changes over development is a non-trivial task. It is no secret that children’s early word usage does not reflect their underlying knowledge. In general, young children’s definitions and, more importantly, their behavior suggests a partial knowledge of the underlying concept even though they can produce the word and appear to fully understand the word (Clark, 1973; Bloom, 2000). Interestingly, tasks assessing this partial knowledge have revealed systematic patterns of word use as children learn the true underlying meanings of words. Around their first birthday, children sometimes show a preference for words to label individual referents and, thus, under-extend a term to other correct referents (Clark, 1973; Kay & Anglin, 1982). For example, a young child may refer to their blanket as *blanky* and refuse to use *blanky* to refer to other blankets. Before their second birthday, children will often over-extend a term, using it to describe inappropriate but often similar referents (Clark, 1973; Rescorla, 1980). For example, children frequently over-extend *dog* to refer to any animal with four legs. In some complicated semantic domains (e.g., kinship, morality), young children continue to over-extend a term for several years. In these cases, children’s over-extensions gradually shift from relying on characteristic features (e.g., a yellow cab with checkered signage is a taxi) to more defining relations (a cab that can be hired for transport is a taxi; Keil & Batterman, 1984; Keil, 1989).

While these behavioral patterns are consistently observed in children’s early word use, it is unclear whether they reflect partial conceptual knowledge (Clark, 1973; Kay & Anglin, 1982), performance limitations—such as retrieving the correct word in the child’s small but rapidly increasing vocabulary (Huttenlocher, 1974; Gershkoff-Stowe, 2001; Fremgen & Fay, 1980), or pragmatic reasoning—such as generalizing a known word when the child’s vocabulary lacks a more appropriate word (Bloom, 1973; Hoek, Ingram, & Gibson, 1986; Barrett, 1986). A major obstacle to teasing apart these alternative hypotheses is the lack of a formalized account of conceptual development predicting children’s word use over time. Specifically, what patterns of word use should we expect as children gather more data? How should these patterns hold cross-linguistically? How do these patterns change as children learn inter-connected conceptual systems (Murphy & Medin, 1985)?

Kinship is an ideal domain to test potentially universal learning mechanisms and to understand the role data plays in acquisition. Kinship systems are present in almost every culture in the world, influencing sociocultural arrangements (e.g., marriage and residence) and social reasoning (e.g., expectations of aid, resource allocation; Mitchell & Jordan, 2021). Therefore, learning and understanding the kinship system one is born into is a vital endeavor for almost every culture in the world. While the importance of kin relationships might vary across cultures, the prominent structure in the world supporting kinship terms, genealogy, is universal.^1^ That being said, kinship systems show remarkable diversity across the languages and cultures of the world both in terms of which relationships get expressed by words (e.g., Murdock, 1949) and the social sanctions for failing to use them correctly. However, despite over a century of data collection and theorizing by anthropologists and linguists, a complete account of kinship systems eludes us. Recent work on efficient communication has shown that two first principles, the trade-off between communicative efficiency and simplicity, can explain at a coarse level the observed diversity in kinship systems (Kemp & Regier, 2012); however, there appears to be no universal principles underlying the evolution of kinship systems as traditionally categorized (Passmore & Jordan, 2020). Therefore, in order to explain evolutionary trajectories, we need fine grained theories and constraints on how kinship systems are structured (Passmore et al., 2021), how different components of kinship terms interact and how kinship terms are acquired. Here, we take the first steps towards providing a formal account of kinship term development that can be used to motivate such theoretical constraints on evolutionary trajectories and can handle the challenge of learning diverse kinship systems.

The goals of this paper are i) to present a rational constructivist framework (Xu, 2007, 2016, 2019) of conceptual development formalized as logical program induction, ii) to evaluate this framework against the literature on children’s patterns of generalization over time—specifically under-extension, over-generalization and the characteristic-to-defining shift, and their order of acquisition. A rational constructivist theory of cognitive development posits that children start with a set of proto-conceptual primitives, which they use to actively construct representations of the world via language and symbol learning, Bayesian inductive learning and constructive thinking (Xu, 2019). We implement a model based on this framework to learn kinship terms, providing the first formal developmental model for kinship term acquisition. The paper is organized as follows: First, we review the empirical literature on kinship term acquisition and computational models of kinship. We then flesh out our model framework and implementation. In presenting the results, we first demonstrate that the model is powerful enough to learn kinship systems of varied complexity based on its input data. We then provide simulations based on informant provided learning contexts to show that the general patterns of children’s word use described above fall out naturally from framing conceptual development as program induction in naturalistic environments. In the process, we present evidence suggesting that children’s early word use might be informative about conceptual development and derive a novel account of the characteristic-to-defining shift. To demonstrate how this model can be used to entertain important theoretical questions about how inductive biases and children’s input drive children’s behavior, we examine the roles of simplicity and environmental input in determining the order of kinship term acquisition. Lastly, we conclude with a discussion of novel predictions and limitations of our account.

## Children’s acquisition of kinship terms

Despite its pervasive influence on our social and cultural interactions, the study of kinship and kinship term acquisition has been minimal (Mitchell & Jordan, 2021). Here, we will focus our review on studies that speak to three specific behaviors: over-/under- extension, characteristic-to-defining shift and order of acquisition. For a thorough review of kinship acquisition, we recommend Mitchell and Jordan (2021), which synthesizes disciplinary approaches and provides a useful developmental toolkit suitable for cross-cultural data collection. To our knowledge, there are no studies designed to directly test patterns of over-/under- extension for kinship terms; however, there are a few lines of work that provide evidence for the phenomena.

First, Piaget (1928)’s study of logical relationships and subsequent replications (Elkind, 1962; Danziger, 1957; Chambers and Tavuchis, 1976; Swartz & Hall, 1972) provide evidence for under-extension. Piaget (1928) conducted targeted interviews with 4 to 12-year-old children to assess their knowledge of logical relations using the sibling concept as a case study. Piaget’s task tested the reciprocity of sibling relationships by soliciting definitions and investigating if children could note the contradiction between the claims that “There are three brothers/sisters in your family” and “You have three brothers/sisters.” Based on his interviews, Piaget proposed that children learning logical relations (like kinship) progress through three stages: egocentric, concrete relational (transitive), abstract relational (reciprocal). An egocentric stage of kinship term use implies a specific pattern of under-extended kinship term use. However, the literature provides sparse and conflicting evidence in support for Piaget’s account. Consistent with Piaget, children (5 to 8 years old) make less mistakes on egocentric concepts (*grandmother*) than other-centric concepts (*granddaughter*) (Macaskill, 1981, 1982). Children (4 to 10 years old) also perform better when questions are framed with respect to themselves (*What is the name of your sister?*) as opposed to another family member (*As for your sister Mary, what is the name of her aunt?;* Greenfield & Childs, 1977). At the same time, equally young children succeed at taking other people’s perspective when providing kin terms (Carter, 1984) and young adopted children (4 to 5-year-olds) have more kinship knowledge than non-adopted children (Price-Williams, Hammond, Edgerton, & Walker, 1977). Moreover, it is unclear that children providing examples of family members when giving a definition reflects an egocentric understanding of kinship as opposed to the use of kinship terms as terms of address (for discussion see Hirschfeld, 1989). Given the limited and conflicting data on egocentric biases in kinship acquisition, we do not directly evaluate our model against the specific egocentric claims in the literature regarding perspective taking. Nonetheless, an initial period of egocentric performance would predict under-extension.

A second line of kinship research lies at the merger of componential analysis in anthropology (Goodenough, 1956) and the semantic feature hypothesis for word learning proposed by Clark (1973). Componential analysis takes up the task of identifying the minimal set of features required to distinguish relevant distinctions in meaning. For example, gender is a required feature of the English kin system because gender is required to distinguish, for instance, mother from father. The semantic feature hypothesis posits that children acquire the semantics of a concept “component-by-component” (Clark, 1973). Thus, developmental studies of kinship acquisition could inform theoretical anthropological studies of componential analysis, especially when multiple sets of components are equally as expressive. As Greenfield and Childs (1977) points out, the pattern of children’s mistakes in an elicitation task is informative about the actual features of meaning children have acquired. These systematic errors are evidence for over-/under-extension. For example, 4 to 5-year-old Zinacantan children’s mistakes never violate the feature that siblings have common parentage; however, half of their mistakes violate gender (i.e., over-extension to incorrect genders), whereas 8 to 10-year-olds never violate common parentage and gender, but violate relative age (over-extension across ages). Therefore, componential analyses that include features for common parentage and gender are more likely than componential analyses that do not. For our purposes, the systematic errors uncovered by the developmental evaluation of componential analyses provides evidence for systematic patterns of over-extension.

The semantic feature hypothesis has also been used to predict the order of acquisition of kinship terms. Haviland and Clark (1974) proposed and found evidence for simplicity to be a driving force in the order of acquisition of English kinship concepts. In their analysis, a relationship between two individuals was considered one feature. Relations that could be explained by appealing to one parent/child relationship (e.g., mother) were learned earlier than relations that required two parent/child relationships (e.g., brother). Similarly terms that required three relationships (e.g., aunt) were learned after those requiring two relationships. Surprisingly, terms that required both a parent and child relationship (e.g., brother) were learned before terms that required the same relationship twice (e.g., grandma). Further support for the semantic feature hypothesis has been found cross-linguistically in definition elicitation studies with German 5-10-years-old children (Deutsch, 1979) and Vietnamese 4 to 16-year-old children (Van Luong, 1986). A similar pattern was reported by Benson and Anglin (1987); however, they explained their data as different amounts of experience with relatives and input frequency of kinship terms. While experience seems to explain differences in adopted children, there was no effect of household size on kinship acquisition (Price-Williams et al., 1977). In general, the extent to which simplicity and experience contribute to the order of acquisition of kinship terms is still an open question, which we directly address in our analysis of order of acquisition effects from model simulations.

To summarize, studies on kinship term acquisition document a protracted developmental trajectory, providing modest evidence for patterns of over- and under- extension in children’s use of kinship terms; although the exact patterns of extension vary across cultures. For example, Bavin (1991) and Greenfield and Childs (1977) find gender over-extensions in Walpiri and Zinacatan children’s kin usage; whereas Price-Williams, Hammond, Edgerton, and Walker (1977)’s study of Hawaiian and the studies on English kin acquisition report no incorrect gender extensions. Interestingly, the children in these studies are well older than the age range where the typical patterns of over- and under- extension described in the introduction are observed. While not all of these studies solicit definitions, the elicitation tasks used are still likely to be challenging for children who have limited verbal ability. Therefore, we should take these patterns with a grain of salt, as young children might not understand the task and older children might lack the verbal ability to articulate their knowledge. Given these limitations, it is unclear that these patterns should fall out of a model of conceptual development as opposed to a model of how children verify semantics or produce labels. This makes it all the more interesting if these patterns do emerge naturally from the inductive learning process, which would suggest that conceptual development may still be contributing to these patterns despite the limitations of the task.

To further ground the possibility of conceptual development giving rise to patterns of over- and under- extension, it is worth mentioning a related field of studies regarding the characteristic-to-defining shift observed in children’s knowledge (Keil & Batterman, 1984; Keil, 1989; Landau, 1982). In Frank Keil’s studies, children are presented with scenarios of a concept—take for example the concept, grandpa—that emphasize either characteristic features but not defining features (e.g., a nice old man who isn’t related to you) or defining features but not characteristic features (e.g., your parent’s evil father). Young children (mean 5;7) are more likely than older children (mean 9;9) to accept a scenario with characteristic features as being true than a scenario with defining features but not characteristic features. Older children are more likely than younger children to accept the scenarios with the defining features of the concept. Remarkably, even some of the older children were not at perfect performance, suggesting that there is significant conceptual development still taking place in kinship beyond the ages in which one typically observes patterns of over- and under- extension. Given this timescale, we argue that children’s over-extensions and under-extensions might actually be due to conceptual development—in particular, rational construction of a logical theory—as opposed to performance-based or pragmatic-based alternative explanations.

In this paper, we implement an ideal learning model using the default assumptions from the rule-based concept learning literature. The model framework is designed to learn a kinship system consistent with the input; however, the model is *not* engineered to match the patterns of behaviors children demonstrate when learning kinship. In other words, the model is unaware of the behaviors children exhibit and, thus, cannot be influenced by explicit knowledge of the evaluation metrics.^2^ We evaluate the model against these patterns of behavior to show that a system for learning program-like structures provides an explanation for the patterns of over- and under-extension behavior we see in children even though it was not engineered to do so. Further, we expand the model by adding assumptions about the learning context (via interviews) and the environmental distribution of data to show that when this model operates under naturalistic contexts and distributions of data, it predicts both a characteristic-to-defining shift and the order of kinship term acquisition that we observe in children.

## Computational models of kinship

From a formal modeling perspective, kinship is an ideal domain for studying how children’s conceptual knowledge develops into the rich rule-like concepts and conceptual systems seen in adult definitions. Kinship easily lends itself to logical representation (e.g., Greenberg, 1949, Wallace & Atkins, 1960). Kinship systems are relational by nature, which makes them interesting because they involve structure, not just similarity. Further, kinship is a test-bed for how inter-related conceptual systems are learned, as adult kinship knowledge suggests inter-related, not independent, concepts for kinship terms.^3^ That being said, most of the previous computational models of kinship has focused on understanding kinship as a mathematical object (e.g., Read, 1984) rather than a cognitive technology (Heyes, 2018; Mitchell & Jordan, 2021).

The earliest computational models of kinship were primarily concerned with automating componential analysis: given a large set of features about each kinship term in a language, what is the minimal set of features required to distinguish the terms (Goodenough, 1956; Lounsbury, 1956)? As Burling (1964) was quick to point out, the componential analysis of a kinship dataset has many possible solutions. Pericliev and Valdés-Pérez (1998) implemented a model to perform componential analysis that finds all possible solutions possessing both the smallest number of unique features and the shortest feature conjunctions required to define all terms. Proving Burling’s point, Pericliev and Valdés-Pérez (1998)’s automated analysis of Bulgarian kinship systems found two equally complex feature inventories that use different features. To complement componential analyses, several behavioral studies used multidimensional scaling techniques to uncover the dimensionality of kinship components and arbitrate between different componential analyses (e.g., Wexler & Romney, 1972, Nakao & Romney, 1984). Recent work in the spirit of componential analysis has taken up the search for kinship universals using optimality theory (Jones, 2010) and Bayesian methods (Kemp & Regier, 2012).

Early connectionist models have used learning kinship as a test case for distributed models of abstract, relational concepts. Hinton (1986)’s family tree task focused on learning an encoding for the family members on a given tree and the relationships between them. The connectionist model received input vectors reflecting an individual on the tree (e.g., *Simba*) and a kinship relationship (e.g., *father*) and output the individuals on the tree who completed the kin relation (e.g., *Mufasa*). The model learned interpretable embeddings for people on the tree, such that semantic features (e.g., gender) could be easily extracted. However, the relationship embeddings were not interpretable and the generalization performance of the model was poor. Paccanaro and Hinton (2001) improved upon the early connectionist models by learning the implicit tree structure behind the training data; however, their model did not fare as well when incorporating held out relations to the model. The model learns the family members and all of the relations on the tree without learning the actual tree structure. Therefore, it’s unclear how well the relations learned will generalize to an entirely new family tree. Importantly, neither connectionist model makes any claims about children’s behavior while learning. Though, Paccanaro and Hinton (2001) points out the most common generalization error was over-extension of sibling terms to include the speaker—i.e., the common failure of Piaget (1928)’s logic problem.

More recent computational models have approached the acquisition of kinship knowledge through a Bayesian relational-learning or theory-learning perspective. The Infinite Relational Model (IRM; Kemp, Tenenbaum, Griffiths, Yamada, & Ueda, 2006) uses the presence or absence of relations between individuals and kinship term use to learn groupings of these individuals and properties shared by the groups, which are diagnostic of the relationship. For example, applying the IRM to data from an Australian kinship system results in groups of individuals that share “diagnostic” kinship relevant feature dimensions such as age and gender. Katz, Goodman, Kersting, Kemp, and Tenenbaum (2008) proposed a generative model similar to the IRM but with a richer representation system based in first order logic, Horn Clause Theories. Their model learns each individual’s kinship relevant properties and the abstract rule governing how those properties give rise to the kinship relation. Katz et al., (2008)’s representation scheme has two advantages over the IRM. First, Horn Clause Theories take into account human reasoning in order to be expressed in the simplest and fewest possible clauses (Kemp, Goodman, & Tenenbaum, 2007). Second, Horn Clause theories are context independent, which allows one’s knowledge of kinship to easily generalize beyond the observed/training data. Similar first order logic representation schemes have been used to analyze the space of all possible kinship systems to identify the pressures that influence which kinship systems are extant in the world (Kemp & Regier, 2012). Surprisingly, extant kinship systems are found at the optimal trade-off between simplicity and communicative efficiency.

Our model builds off the intuitions of the Bayesian models. Following Katz et al., (2008), we adopt the use of a context-independent representation scheme. Like this model and others (Kemp, 2012; Haviland and Clark, 1974), our model also incorporates a pressure for simplicity. However, our approach will depart from past models in two ways. First, our representation scheme is inspired by set theory instead of e.g., Horn clauses,^4^ which provide poor fits to adult induction and generalization behaviors (Piantadosi, Tenenbaum, & Goodman, 2016). Operating over extensional concepts like sets provides more affordances as a representation scheme (e.g., generating members of those sets or possible word referents) than intensional representations like logic. Second, we aim to provide not only a proof of learnability but an evaluation of the full developmental trajectory of concepts, including the common behavioral patterns of mistakes children display.

## The approach: Concept induction as program induction

The basic premise of our approach is that conceptual knowledge can be likened to a computer program (e.g., Lake, Salakhutdinov, & Tenenbaum, 2015; Piantadosi & Jacobs, 2016; Goodman, Tenenbaum, & Gerstenberg, 2015; Goodman, Tenenbaum, Feldman, & Griffiths, 2008; Rule, Tenenbaum, & Piantadosi, 2020; Piantadosi, Tenenbaum, & Goodman, 2012), at least following a computational level of analysis (Marr, 1982). This metaphor capitalizes on several similarities between programs and concepts. First, both programs and concepts are relational in nature. Concepts are defined in terms of both their extension and their relations between other concepts (e.g., dog and wolf share common origin). Whereas programs can be mathematically defined in terms of input/output relations. Second, conceptual development and program induction both emphasize the dynamic nature of knowledge. When a young child originally pieces together a concept, it can be thought of as chaining inferences about what underlying features or relationships are good approximations to the concept’s true meaning. Similarly in program induction, the model chains inferences about what underlying base functions or relationships between base functions are good approximations to the program’s desired output. Lastly, concept and program induction can both result in many intensionally distinct representations that are extensionally equivalent. The principles that a programmer might use to choose between two equivalent representations (e.g., simplicity, minimal hidden structure and ease of deployment; see (Rule et al., 2020)) are the same principles we see in children’s explanations (e.g., Walker, Bonawitz, & Lombrozo, 2017; Johnston, Johnson, Koven, & Keil, 2016).

## The Model

For our ideal learner model, we must specify three components: a hypothesis space over concepts ${\mathscr{H}}$, a prior over hypothetical concepts *P*(*h*) for $h \in {\mathscr{H}}$ and a likelihood function *P*(*d*|*h*) to score the hypothesis according to the data *d*. The hypothesis space reflects the cognitive architecture supporting learning. For example, our hypothesis space consists of compositional functions over family trees. The prior reflects the inductive biases that we suspect children bring to a learning task.

For implementing our model, we must also specify how we simulate data for our learning analyses. Here, a data point *d* is a collection of four objects: a *speaker*, who uses a *word* to refer to a *referent* in a *context* (detailed further below). We model learning as the movement of probability mass across a hypothesis space as a function of observing data. Following Bayes rule, the posterior probability of a hypothesis *h* after observing a set of data points *D* is:

1 $$ P(h|D) \propto P(h) {\prod}_{d \in D} P(d|h). $$

We will discuss each component in turn.

### Hypothesis space

Constructing the hypothesis space over possible programs involves specifying primitive^5^ base functions for kinship that are available to the learner and the method by which these functions compose to form hypotheses. The use of semantic primitives has a rich tradition in linguistics and anthropology^6^ (e.g., Goodenough, 1956, Lounsbury, 1956, Wierzbicka, 2016). In our model, we specify several types of base functions—tree-moving functions (parent, child, lateral), set theoretic functions (union, intersection, difference, complement), observable kinship relevant properties (generation, gender, co-residing adult), and variables—the speaker (denoted X) and the individuals in the context. Tree-moving functions take as argument a reference node in a tree and return a set of nodes satisfying a specific relationship on the tree. As justification for including tree primitives, we note that affording these abilities to children is in line with the proposal from comparative cognition that these relations are innate biological predispositions^7^ (Chapais, 2014) and a common assumption in the literature (e.g., Haviland & Clark, 1974). Set functions allow for first-order quantification, which has been shown to be relevant for adults’ concept acquisition (Piantadosi et al., 2016; Kemp, 2012). Infants can discriminate between gender (e.g., Quinn, Yahr, Kuhn, Slater, & Pascalis, 2002) and preschoolers can discriminate age (Edwards, 1984). We assume that children can compute functions from a speaker’s perspective. We note that these are all non-trivial assumptions, but we have made them based on our best guess about children’s abilities. However, it is simply an empirical question—left for future work—what resources children have before they begin acquiring these terms.

We compose the base functions using a probabilistic context free grammar (PCFG; see Table 1) following (Goodman et al., 2008; Piantadosi et al., 2012; Ullman, Goodman, & Tenenbaum, 2012). Briefly, a PCFG is a set of rewrite rules which describe how functions can compose while defining a potentially infinite space of possible compositions. For example, the composition leading to the concept of grandpa would require applying the male rule, parent rule, parent rule and speaker rule, resulting in the program: *male*(*parent*(*parent*(*X*))). A program can then be evaluated in a context to produce a set of possible referents.^8^ The use of a PCFG is meant to formalize the space of possible hypotheses, not necessarily to provide an algorithmic model of how people search this space. In addition to defining the hypothesis space, the PCFG also provides the prior probability distribution over that space. In this distribution, we weight each rule equally as likely with two exceptions. First to prevent infinite recursion when generating hypotheses, the speaker, X, is weighted ten times as likely as the other rules. Second, we divide the weight for concrete referents equally among the individuals in our context (detailed below).

**Table 1:** The Probabilistic Context Free Grammar (PCFG) specifying the base functions and the rewrite rules that govern their composition. Each hypothesis starts with a SET symbol and there are 37 concrete referents in our learning context

SET $\xrightarrow {1}$ union(SET,SET)	SET $\xrightarrow {1}$ parent(SET)	SET $\xrightarrow {1}$ generation0(SET)	SET $\xrightarrow {1}$ male(SET)
SET $\xrightarrow {1}$ intersection(SET,SET)	SET $\xrightarrow {1}$ child(SET)	SET $\xrightarrow {1}$ generation1(SET)	SET $\xrightarrow {1}$ female(SET)
SET $\xrightarrow {1}$ difference(SET,SET)	SET $\xrightarrow {1}$ lateral(SET)	SET $\xrightarrow {1}$ generation2(SET)	SET $\xrightarrow {1}$ sameGender(SET)
SET $\xrightarrow {1}$ complement(SET)	SET $\xrightarrow {1}$ coreside(SET)	SET $\xrightarrow {\frac {1}{37}}$ concreteReferent	SET $\xrightarrow {1}$ all SET $\xrightarrow {10}$ X

We note that here we do *not* include recursive calls in our PCFG, meaning, for instance, that we cannot represent grandpa as *father*(*parent*(*X*)). In Appendix C, we provide a version of the model that uses recursion, but we note that it is computationally more difficult to implement and also makes identical predictions in many formulations.

### Simplicity Prior

One advantage of using a PCFG is that it builds in a natural prior towards simplicity. Hypotheses that compose more rules are less probable than hypotheses that compose less rules. We motivate this bias towards simplicity in several ways. First, adults have been shown to learn logically simpler concepts faster than complex concepts (Feldman, 2003, 2000; Goodman et al., 2008; Piantadosi et al., 2016). Second, children prefer simpler explanations over more complex explanations (Lombrozo, 2007; Bonawitz & Lombrozo, 2012; though see Walker et al., 2017). In language learning, simplicity has been suggested as a guiding principle (Chater & Vitányi, 2007) that solves the logical problem of acquisition. In kinship specifically, simplicity has previously been proposed as the driving factor behind the order of acquisition of kinship terms (Haviland & Clark, 1974). In a global analysis of all possible kinship systems, simplicity is a good predictor of which kinship systems are actually observed in the languages of the world (Kemp & Regier, 2012). Therefore, we believe simplicity is an important inductive bias for our model. The model exhibits a simplicity bias because the PCFG scores the probability of a hypothesis as a product over rules (thus each additional rule lower’s a hypothesis’ prior):

2 $$  P(h) = {\prod}_{r \in h} P(r), $$

where *r* reflects a single use of a rule from Table 1. Our measure of simplicity has recently been empirically validated for explaining adult acquisition of kinship terms (Smith, Frank, Rolando, Kirby, & Loy, 2020).

### Size principle likelihood

The last component of the model to be specified is the method of scoring the probability of the data under each hypothesis, *P*(*d* ∣ *h*). Based on past research with adults (Tenenbaum, 1999; Tenenbaum & Griffiths, 2001), children (Xu & Tenenbaum, 2007a, 2007b; Lewis & Frank, 2018) and infants (Gweon, Tenenbaum, & Schulz, 2010), we use a size-principle likelihood. This comes from the notion that the data we observe is generated from a structure in the world (i.e., strong sampling) as opposed to randomly generated (i.e., weak sampling). Our implementation marginalizes over two possible ways a learner might think the data was generated. First, the data might be generated according to the learner’s current hypothesis. For a given context, there is a finite set of data points that a learner expects to receive. Following a size principle likelihood, data points are sampled randomly from these expected data points: $\frac {1}{|h|}$, where |*h*| is the number of unique data points (i.e., speaker-word-referent combinations) that a learner expects to see in a given context. Second, a learner might think that a data point was generated by noise—i.e., randomly mapping a speaker, word and referent. In this case, the probability of a data point is given by $\frac {1}{|\mathcal {D}|}$, where $|\mathcal {D}|$ reflects the number of all possible speaker-word-referent pairs in a given context. Our likelihood mixes these two generative processes together by adding a new parameter *α* reflecting the reliability of the data. At high values of *α*, the learner thinks that most of the data is being generated by their conceptual hypothesis; whereas at low values of *α*, the learner thinks the data they see is randomly generated. Combining both of these processes, our likelihood function is given by:

3 $$ P(d|h) = \delta_{d \in h} \cdot \frac{\alpha}{|h|} + \frac{1-\alpha}{|\mathcal{D}|}, $$

where *δ*_*d*∈*h*_ is 1 when the speaker-word-referent *d* is true under *h*, and 0 otherwise. This likelihood, strong sampling, is a powerful likelihood function that can lead to convergence on the true generative process of the data from positive evidence alone (Tenenbaum, 1999) and even in the presence of significant noise (Navarro, Dry, & Lee, 2012).

Having a noisy process directly accounts for an attribution problem that every learner faces: was this data point generated from some true structure in the environment (i.e., is it reliable and valid?) or was this data point possibly a mistake? Social learners are sensitive to the reliability of their instruction (Birch, Vauthier, & Bloom, 2008; Jaswal & Neely, 2006; Koenig & Harris, 2005; Pasquini, Corriveau, Koenig, & Harris, 2007, cf.; Gweon & Asaba, 2018) and language learners have been shown to filter their input to focus on explaining a subset of their data (Perkins, Feldman, & Lidz, 2017). This reliability filtering allows us to account for any issues the learner has mapping words to referents, including the significant challenges of resolving allo-centric reference, the mapping for genitive (e.g., *your daddy*) or alter-centric (e.g., a mother saying *daddy is coming*) uses of kinship terms. If the learner cannot successfully map words and referents, they should act as if their data is being generated unreliably. In Appendix A, we check that our results are robust to different implementations of a noisy size principle likelihood—i.e., values of *α*.

### Environmental assumptions for simulating data

Ideally, we should be using this model to predict empirical measures of word understanding or use. Unfortunately, there are no existing data sets that either quantitatively measure children’s kinship term use or span the nine years of a single child’s experience with kin and kinship terms with the required detail to fully specify the input data for the learning model. As a result, we adopt a simulation approach to generate predictions about children’s word use from basic assumptions about what data children see. We then qualitatively compare our predictions to the trends in children’s behavior reported in the literature.

**Fig. 1:**
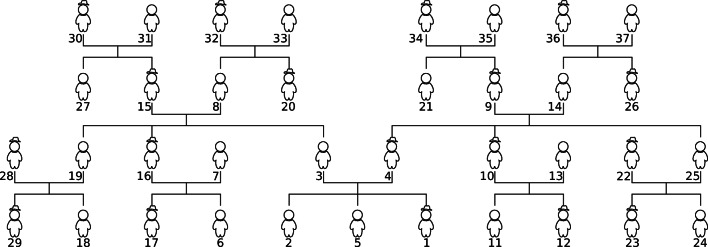
Family tree context for our simulations. Connections above figures reflect parent–child relationships. Connections under figures reflect lateral/spousal relationships. Men are denoted with *hats*. *Numbers* reflect the rank order of the amount of interaction a learner (i.e., 1) has with the other individuals on the tree

For our model, a data point has four components, the speaker, the word, the referent and the context. The context is a family tree, which contains each member of the family, their parent, child and lateral connections and their gender (see Fig. 1). To simulate the data for learning, we first generate all true possible data points given the target word and the context. We then sample data points from the true set with probability *α* or construct a random data point with probability 1 − *α*. For all analyses reported in the paper, *α* was set at 0.90.^9^ In simulating the data this way, we make two simplifying assumptions. First for tractability, we only sample the data from one family tree even though children are exposed to multiple family trees. To ensure the learner received adequate data that might be obtained by children across trees, our tree context spans more of the possible familial relations than our informant provided family trees. To ensure our learner does not over-fit to our context, we vary the speaker across data points, resulting in 29 different perspectives of the same tree. We describe where this assumption influences our conclusions. Second, for convenience, we assume that the referent is computed with respect to the speaker. This is not an assumption about children’s learning but a necessary assumption for formalization in the absence of natural data with explicit annotation of the kinship relation. Ideally, our model would calculate the relation after the appropriate reference person has been identified via perspective taking and/or linguistic processing (e.g., genitives).

## Model evaluation

While our model links data, inductive biases and conceptual representations, there are many ways these could be related to children’s behavior. For example, in a comprehension task, a child might have a context containing several individuals and their goal is to point to *uncle*. Our model provides a posterior distribution over what this word might refer to, but there are many ways a child might use that posterior to respond. For example, they could just select the maximum probability referent. They could sample from the referents based on each individual’s probability. They could perform pragmatic reasoning as in a rational speech act model (Frank & Goodman, 2012) and condition on other words in their vocabulary to adjust these probabilities. Alternately, children might sample a single hypothesis (e.g., Bonawitz, Denison, Gopnik, & Griffiths, 2014; Medina et al., 2011) either based on the posterior probability or weighted by the value of computation as in a feed-forward pragmatics (Ferreira, 2019; Lieder & Griffiths, 2017). Here, we present our results marginalizing over the posterior, meaning we show posterior average responses, which might correspond to subject average responses under the assumption that subject behavior matches the probability estimated by the model. We further discuss how mutual exclusivity will emerge from a rational speech act linking hypotheses in Appendix D.

We divide the model evaluation into three sections: Model Outcomes, the Characteristic-to-Defining Shift and Order of Acquisition. In Model Outcomes, we first check that the model successfully learns the conventionally agreed upon extension for each kinship term in finite amounts of data. We conduct this analysis using four different kinship systems: Pukapukan, English, Turkish and Yanomamö. We then take a closer look at how the model behaves locally at the outset of learning to demonstrate how children’s early preference for concrete reference—i.e., under-extension, naturally follows from the process of induction with few data points. Afterwards, we look at how the broad pattern of over-generalization falls naturally out of the process of induction when trading off simplicity and fit to the data. Our primary finding is that an inductive learning model with program-like representations and biases for simplicity and strong sampling will accurately learn a kinship system consistent with its input in finite amounts of data and predict under-/over-extension as a consequence of insufficient data.

In Characteristic-to-Defining Shift, we augment the model’s hypothesis space, allowing rules based on characteristic features (e.g., uncle : *union*(*big*,*strong*)). We first replicate our previous analyses using simulations based on naturalistic learning contexts—i.e., informant provided family trees. For each word learned by each informant, we demonstrate the characteristic-to-defining shift. We discuss how the characteristic-to-defining shift arises from properties of the learning context and under what circumstances we would predict to see a characteristic-to-defining shift. Our primary finding is that the characteristic-to-defining shift emerges from an inductive learning mechanism in naturalistic environments without appealing to a discontinuity in representation space or learning process or the development of abstraction.

In Order of Acquisition, we return to an open question in the kinship acquisition literature: is the order of acquisition driven by experience or the conceptual complexity of the kinship relations? We evaluate the model predicted order of English kinship acquisition against the empirically observed order of concept acquisition in children. We illustrate that while the simplicity of the minimal description length correct kinship concepts aligns with the observed order of acquisition in children, the model does not predict acquisition in that order. Inspired by accounts of children’s experience with kin relations (Benson & Anglin, 1987), we simulate several plausible data distributions based on kin experience and find that the order of acquisition is more likely driven by *both* conceptual simplicity and naturalistic data distributions rather than by conceptual simplicity alone. Of course, fine-grained household data will be invaluable for addressing the question of experience and collecting such data will require a significant, concerted effort.

### Model outcomes

#### The model learns typologically diverse systems as input varies

**Fig. 2:**
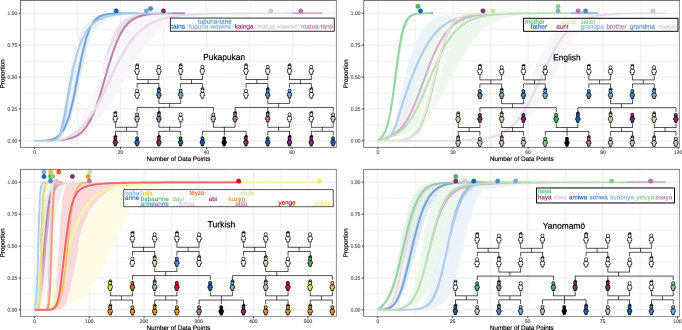
Average lexicon posterior-weighted accuracy for each word as a function of data points of that word. *Shaded region* denotes 95*%* bootstrapped confidence intervals. *Insets* show the color-coded extension of the terms

We first simulated data for four kinship systems that vary in descriptive complexity and are common in the languages of the world: Pukapukan, English, Turkish and Yanomamö. Extensions for the kinship terms of these languages are provided in the insets of Fig. 2 and Table 2. The Pukapukan kinship system is six kinship terms that are fully described by generation and gender. The English kinship has nine terms that require representing parent/child relations. The Turkish system has fourteen kinship terms with high specificity in the first generation. In addition to requiring tree moving functions, the Turkish requires separating paternal and maternal brothers and sisters and their spousal relationships. The Yanomaö system has eight kinship terms with a notable distinction between cross-cousins—i.e., the children of parents’ opposite-sex siblings, and parallel-cousins—i.e., the children of parent’s same-sex siblings. Capturing this distinction between cousins is possible with the same set of primitives required for Turkish; however, the hypothesized concepts would require many primitives to be composed. The complexity required for this distinction, however, may be mitigated by its importance to Yanomamö society, which follows strict bilateral cross-cousin marriages and maintains patrilocal residence. When we incorporate this important sociocultural information into the hypothesis space via the coresidence primitive, the complexity of Yanomamö kinship concepts decreases.^10^

**Table 2:** The maximum-a-posterior (MAP) hypotheses after learning

	Word	Extension	MAP Hypothesis
Pukapuka	*kainga*^*‡*^	z, pgd, ped	difference(generation0(X), sameGender(X))
	*matua-tane*	pb	male(child(parent(parent(X))))
	*matua-wawine*	pz	female(child(parent(parent(X))))
	*taina*^*‡*^	b, pgs, pes	intersection(generation0(X), sameGender(X))
	*tupuna-tane*	pf	male(child(parent(parent(parent(X)))))
	*tupuna-wawine*	pm	female(child(parent(parent(parent(X))))
English	*aunt*	pz, pgw	female(difference(generation1(X), parent(X)))
	*brother*	b	male(child(parent(X)))
	*cousin*	pgc, pgec	difference(generation0(X), child(parent(X)))
	*father*	f	male(parent(X))
	*grandma*	pm	female(parent(parent(X)))
	*grandpa*	pf	male(parent(parent(X)))
	*mother*	m	female(parent(X))
	*sister*	z	female(child(parent(X)))
	*uncle*	pb, pgh	male(difference(generation1(X), parent(X)))
Turkish	*abi*	b	male(child(parent(X)))
	*abla*	z	female(child(parent(X)))
	*amca*^*‡‡*^	fb	intersection(sameGender(*fabio*), difference(child(parent(male(parent(X)))), parent(X)))
	*anne*	m	female(parent(X))
	*anneanne*	mm	female(parent(female(parent(X))))
	*baba*	f	male(parent(X))
	*babaanne*	fm	female(parent(male(parent(X))))
	*dayi*	mb	male(child(parent(female(parent(X)))))
	*dede*	pf	male(parent(parent(X)))
	*eniste*	pgw	intersection(lateral(child(parent(parent(X)))), male(complement(parent(X))))
	*hala*	fz	female(child(parent(male(parent(X)))))
	*kuzen*	pgc, pgec	difference(generation0(X), child(parent(X)))
	*teyze*	mz	difference(difference(female(generation0(female(parent(X)))),X),parent(X))
	*yenge*	pgh	difference(female(generation1(X)),union(child(parent(parent(X))),parent(X)))
Yanomamö	*amiwa*	z, fbd, mzd	female(child(coreside(X)))
	*eiwa*	b, fbs, mzs	male(child(coreside(X)))
	*haya*	f, fb	male(coreside(X))
	*naya*	m, mz	female(coreside(X))
	*soaya*	mb	male(difference(generation1s(X), coreside(X)))
	*soriwa*	mbs, fzs	difference(male(generation0(X)), child(coreside(X)))
	*suaboya*	mbd, fzd	female(difference(generation0(X), child(coreside(X))))
	*yesiya*	fz	difference(female(generation1s(X)), coreside(X))

f:father, m:mother, p:parent, s:son, d:daughter, c:child, b:brother, z:sister, g:sibling, h:husband, w:wife, e:spouse ^‡^ The extension is provided with regards to a male speaker. For a female speaker, swap the two words. The MAP hypothesis will compute the correct extension regardless of speaker’s gender. ^‡‡^ The MAP hypothesis for *amca* makes use of *Fabio*, the individual ranked 29 in Fig. 1 in order to construct the set of all men in the context

Figure 2 shows the predicted learning curves for each kinship term in Pukapuka, English, Turkish and Yanomamö. The *x*-axis shows the number of data points for each word observed by the child. Note the differences in scale across languages. The *y*-axis is the probability that a learner has acquired the conventionally-aligned upon meaning of that term—i.e., extends the term appropriately. The shaded region represents the 95% bootstrapped confidence interval. The line for each word is color coded to match the word’s extension in the inset. Table 2 provides the maximum-a-posteriori hypotheses learned for each kinship term.

While different languages favor different base functions and require differing levels of complexity, the same model successfully learns a set of computations equivalent to the conventional kinship systems for each of these languages based solely on differences in data input. Further, the model learns these kinship systems with fairly few data points, on average between 30 and 50 data points per word learned. As a back-of-the-envelope feasibility check, if we assumed that an effective data point, following Mollica and Piantadosi (2017) arrives on average once every two months, we would expect the mean age of acquisition to be between 5 and 8.333 years after children start attending to kinship terms. This is consistent with the observed protracted trajectory discussed in our review of empirical acquisition phenomena. We discuss the differences between this model’s predicted acquisition order and children’s empirical order for English in the Order of Acquisition section. Unfortunately, we could not find empirical data for the order of acquisition of Pukapukan, Turkish and Yanomamö kinship terms.

#### The model shows an early preference for concrete reference

**Fig. 3:**
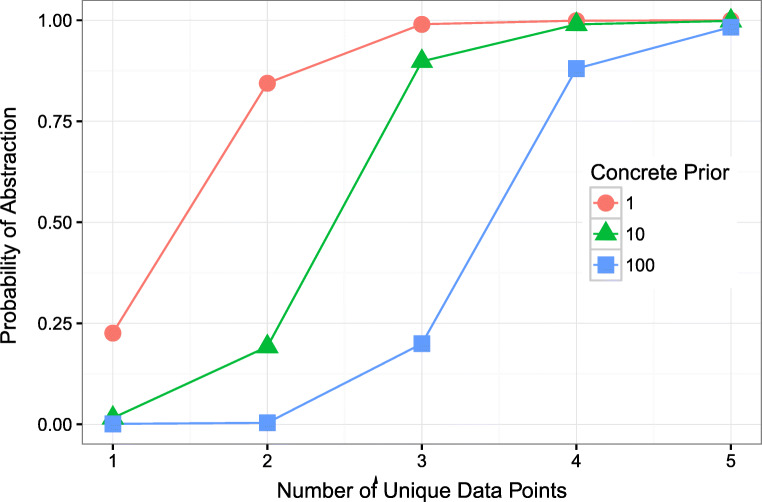
Probability of using abstraction as a function of unique data points at several different prior strengths for concrete reference. At higher prior values of concrete reference, the rise in the probability of abstraction is shifted to require more unique data points

Young children typically restrict their word usage to refer to particular individuals, or concrete referents, rather than draw abstractions over individuals (Clark, 1973; Kay & Anglin, 1982). This pattern naturally falls out of our model’s push to explain the data when there are few unique data points, suggesting that the preference for using concrete reference is driven by the data observed rather than by inductive biases of the model. To look at the model’s preference for concrete reference, we highlight a single concept, uncle, and focus on the first five unique data points that the model observes (see Fig. 3). The *x*-axis in Fig. 3 reflects the number of unique data points (i.e., distinct referents) for a word. The *y*-axis represents the probability the model uses abstraction to move away from concrete reference. With no inductive bias favoring concrete reference (red circles), the model initially favors concrete referents approximately 75*%* of the time. As more unique data points are observed, the model quickly switches to abstracting away from concretes referents.

This behavior is observed because at low data amounts, the best hypothesis that explains the data is a concrete referent. For example, if you only ever encounter the word *uncle* to refer to Joey the best hypothesis is to think that uncle just denotes Joey. As the model observes more data, it becomes too complicated to store all the possible referents and so the model adopts simpler rules that abstract away from the data. This movement away from concrete reference after seeing two unique referents might seem fast, given that children are often willing to provide multiple example referents before their definitions use abstraction. One possibility is that children are using kinship terms as a form of address. Therefore, their provision of referents is not a reflection of their kinship concept but of their terms of address for specific people, which extends beyond kin (e.g., *teacher*). Another possibility is that children have an inductive bias favoring concrete referents. In Fig. 3, we plot the probability of abstraction when the model has a 10:1 (green triangles) and 100:1 (blue squares) bias for using concrete reference as opposed to abstraction. As the bias for concrete referents increases, more unique data points need to be observed before the model favors using abstraction. Whereas, if children memorize terms of address like proper names, the number of unique referents should not influence their use of kinship terms. Given the importance of unique referent amount to our model, future work may directly tease apart the conceptual origin (genealogical vs an address-system) for kin terms and when children switch from learning one structure to the other by investigating children’s sensitivity to unique referents in artificial kinship learning tasks.

#### The model predicts over-extension

**Fig. 4:**
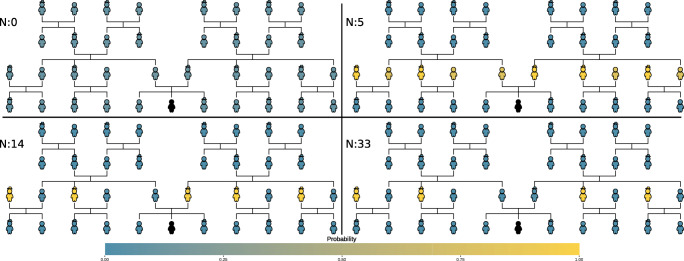
The posterior probability that each person on the tree is an uncle of the learner (*in black*) at various data amounts. *Yellow* (lighter color) indicates high probability and *blue* (darker color) indicates low probability

Older children embrace abstraction; however, the rules they learn often over-extend a word to include incorrect referents (Clark, 1973; Rescorla, 1980). For example, all women might be recognized as *aunts*. Unlike under-extension, which is driven by the local data distribution at the onset of learning, over-extension is a global behavior of our model. The model not only predicts over-extension but predicts specific patterns of over-extension as a function of the data it has observed and the base functions supporting the hypothesis space. For example, Fig. 4 shows the model’s predicted pattern of use for the term *uncle* conditioned on a learner, represented in black, at different amounts of data. At low amounts of data, everyone in the context is equally unlikely to be denoted by uncle. Within the first 5 data points, the model extends the term to all members of the learner’s parent’s generation (which is a base function). By 14 data points, the model has narrowed that down to only the men of that generation (which is the composition of two base functions). Near 33 data points, the model’s extension looks very adult-like; however, it is important to note that the model still needs to tease apart several different hypotheses that might make unwarranted predictions if the context was to vary. In fact, the model does not come to learn the context-invariant concept of uncle until around 45 data points.

Over-extension in the model falls out of the interaction between the size-principle likelihood and the base functions supporting the hypothesis space. The size principle likelihood posits that it is better to predict both observed and unseen data than to fail to predict observed data. Therefore, once the model has exhausted simple concrete hypotheses, it begins to abstract but it prefers to abstract using base functions that cast wide nets over referents—i.e., predicting many referents. The model will shift from these simple wide-reaching hypotheses to narrower hypotheses as it observes more data that can be explained better by a more complicated hypothesis. As a result, the patterns of over-extension should be predicted by base functions and compositions of base functions that increasingly approximate the true concept. We provide model predictions of the over-extension pattern for each kin term in supplemental material as an illustration.^11^

**Fig. 5:**
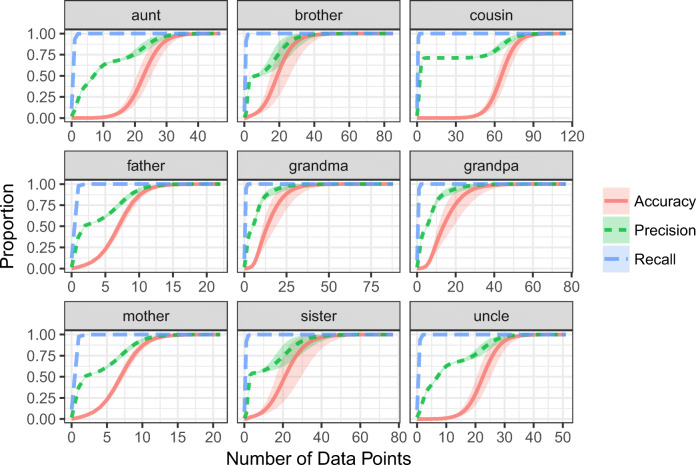
Average lexicon posterior-weighted accuracy, precision, and recall for each word as a function of data points. Recall greater than precision is a hallmark of overgeneralization. *Shaded regions* represent 95*%* bootstrapped confidence intervals

We can also compare the model’s posterior weighted recall and precision. Recall is the probability of comprehending a word when it is used correctly. With a wide enough hypothesis, a learner will accept all of the correct uses of a word—although they will often accept incorrect uses of a word as well. Precision is the probability of producing a correct referent given the learner’s current hypothesis. For example, if the learner had the correct definition of *uncle*, they would produce only and all the correct uncles and so precision would be 1.0. If the learner had a current hypothesis that over-generalized, they would produce correct uncles only a fraction of the time, even if their current hypothesis contained all of the real uncles. As a result, precision would be less than one. To visualize the presence of over-generalization, we use an *F*_1_ score plot to compare posterior weighted precision to posterior weighted recall. Greater recall than precision is a hallmark of over-extension. Fig. 5 illustrates this signature pattern of over-extension for each word in English.^12^ The variation in precision is driven by the specific patterns of over-extension predicted by the model (see supplemental materials for model predictions). We will discuss order effects in the Order of Acquisition section.

### The characteristic-to-defining shift

The characteristic-to-defining shift is a prevalent pattern of children’s over-extension. Young children are more likely to over-extend using characteristic features (e.g., robbers are *mean*) as opposed to defining features (e.g., robbers *take* *things*). While the characteristic-to-defining shift is commonly observed, the process which leads to it is unclear. One possibility is that the characteristic-to-defining shift is a stage-like transition that occurs in the representational system (Werner, 1948; Bruner, Olver, & Greenfield, 1966). For example, the shift could be explained by a transition from representing concepts holistically—i.e., using all the features of objects, to representing concepts analytically—i.e., narrowing in specific relevant features of objects (Kemler, 1983). Neural network models of conceptual classification inherently capitalize on this idea when demonstrating a shift (e.g., Shultz, Thivierge, & Laurin, 2008). Another possibility is that there is a change in the mechanism by which one learns concepts. For example, concept learning might change from storing exemplars to constructing prototype or rule-based representations. These hypothetical changes in representation or processing might be maturational in nature, such as the development of abstraction (Piaget & Inhelder, 1969). Alternately, they may be driven by inductive inference mechanisms operating over observed data, à la rational constructivism.

From the outset, we can narrow down this space of theoretical hypotheses. The characteristic-to-defining shift is most likely a function of data, not maturation (Keil, 1983). One prediction of a maturational-shift is that at a single time-point, children should represent all words using characteristic features or defining features, whereas a data-driven shift predicts that both adults and children should have more exemplar-based representations in unfamiliar domains, and more rule-based representations in familiar domains. The former does not explain children’s behavior: children seem to possess characteristic representations and defining representations of different words at a single time point. The prediction of the latter—that individuals have more exemplar-based representations in unfamiliar domains and more rule-based representations in familiar domains—is observed in children (Chi, 1985) and in adults (Chi, Feltovich, & Glaser, 1981).

All of the aforementioned explanations for the characteristic-to-defining shift require a discrete shift in representation or process. However, no model has tested whether a characteristic-to-defining shift could be a natural by-product of the continuous data-driven construction of concepts, as found in our model, and similar to conceptual garden-pathing (Thaker, Tenenbaum, & Gershman, 2017) or learning traps (Rich & Gureckis, 2018). We expect our model to demonstrate a characteristic-to-defining shift only if the characteristic features of the people in the context are informative but imperfect in their ability to capture the underlying concept (by denoting the proper referents). If the characteristic features accurately capture a concept, the model should never shift from favoring characteristic hypotheses to defining hypotheses. On the contrary, if the characteristic features are uninformative, and thus poor at capturing a concept, our model should favor defining hypotheses, predicting either no shift or an implausibly rapid shift from characteristic-to-defining hypotheses.

Because these model predictions depend critically on the types of characteristic features present in real data, it is not straightforward to use simulation to create these features because the outcome will depend on the nature of the simulated data. Instead, we collect data about the characteristic and logical relationships of real people to test if natural data will contain features within the range of informativity that will show a characteristic-to-defining shift.

#### Data collection

**Fig. 6:**
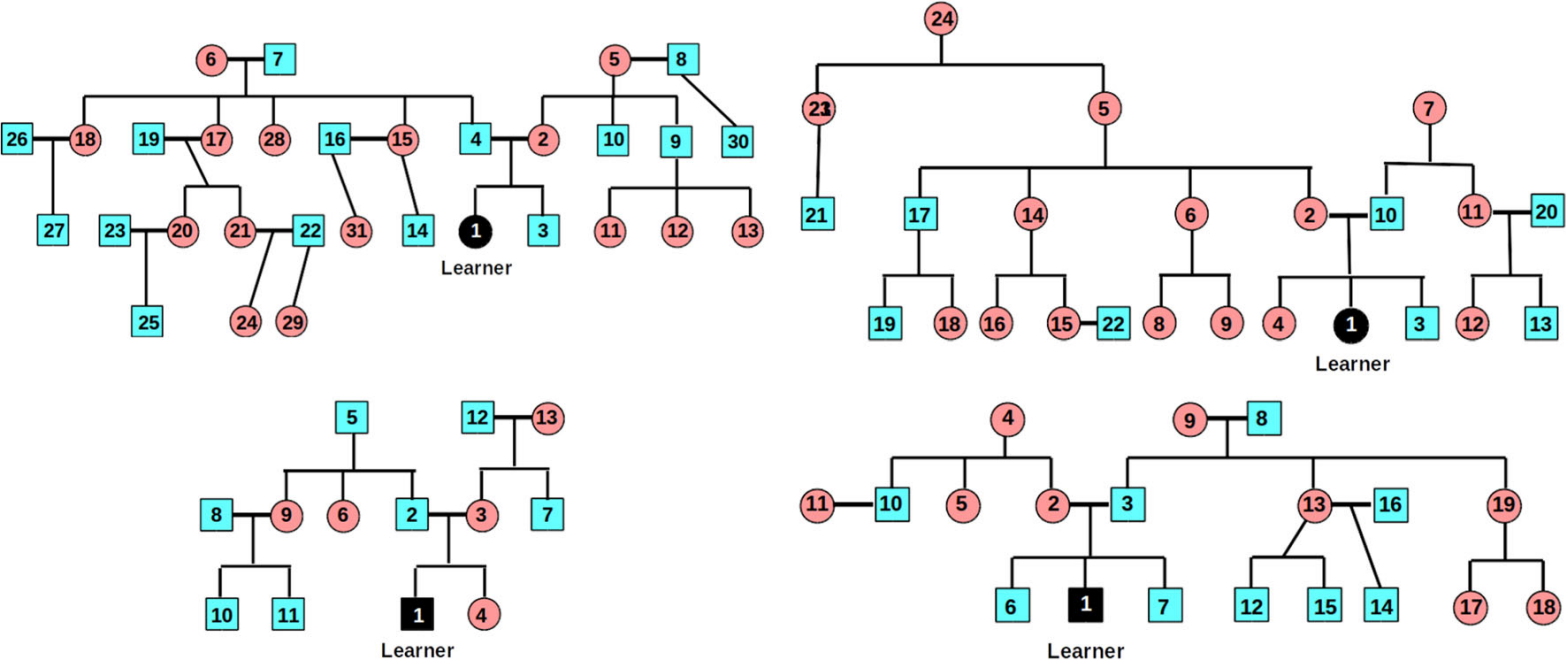
Distance-ranked family trees from informants. *Circles* represent women; *squares* men. *Bold lateral lines* denote spousal relationships. Informant 1 (*top left*) provided 107 unique features; Informant 2 (*top right*) 88; Informant 3 (*bottom left*) 92; and Informant 4, 59

We asked informants to provide us with information about their family trees. Four informants, who were unaware of our purpose, drew their family tree, ranked each family member in terms of how frequently they interacted with them as a child (see Fig. 6), and provided ten one-word adjectives for each family member. For each informant, the unique adjectives were used to construct a binary adjective by family member feature matrix. Each informant was presented with the feature matrix and asked to indicate if each feature applied to each family member. Informants made a response to every cell of the matrix: zero if the feature did not apply; one if the feature did apply. The informants provided between 59—107 (*M* = 86.5) unique features including both experiential features (e.g., *strict*) and perceptually observable features (e.g., *blonde*).^13^

#### Implementation details

To model the characteristic-to-defining shift, we used the informant provided tree contexts to simulate data for learning. For each informant, we used their solicited features to augment the hypothesis space (Table 1) with the rules in Table 3. As a result, the hypothesis space now includes characteristic hypotheses that return the set of individuals the informant labeled as having that feature. For example, *outgoing(Yes)* generates the set of individuals in the context marked as outgoing and *union(small(Yes), outgoing(Yes))* returns the set of individuals in the context marked both small and outgoing. This augmented grammar allows us to model learners as comparing characteristic (elicited) features vs. defining (logical, as above) features and compute the probability of each type of hypothesis.

**Table 3:** Additional rules for the PCFG in Table 1. Now, each hypothesis starts with a START symbol

START $\xrightarrow {1}$ SET	FSET $\xrightarrow {1}$ union(FSET,FSET)	FSET $\xrightarrow {1}$ intersection(FSET,FSET)	FSET $\xrightarrow {1}$ feature(VALUE)
START $\xrightarrow {1}$ FSET	FSET $\xrightarrow {1}$ complement(FSET)	FSET $\xrightarrow {1}$ difference(FSET,FSET)	VALUE $\xrightarrow {1}$ {Yes—No}

#### Results and discussion

It should be noted that the informant provided contexts are smaller/sparser than the context used in our previous analyses (Fig. 1). As a result, the model might not see types of data that are required for learning the context-invariant kinship concept.^14^ Nevertheless, it does not influence our ability to observe a characteristic-to-defining shift. While the MAP hypotheses are not context-invariant, the model always learns a program that selects the individuals consistent with the observed data. In Appendix B, we provide *F*_1_ plots for all informants and English kinship terms, and discuss the situations in which the model does not learn the “correct” concept for a kin term. Our failure to learn all terms from these simulations suggest that egocentric kinship data is not always sufficient for learning kinship terms.

**Fig. 7:**
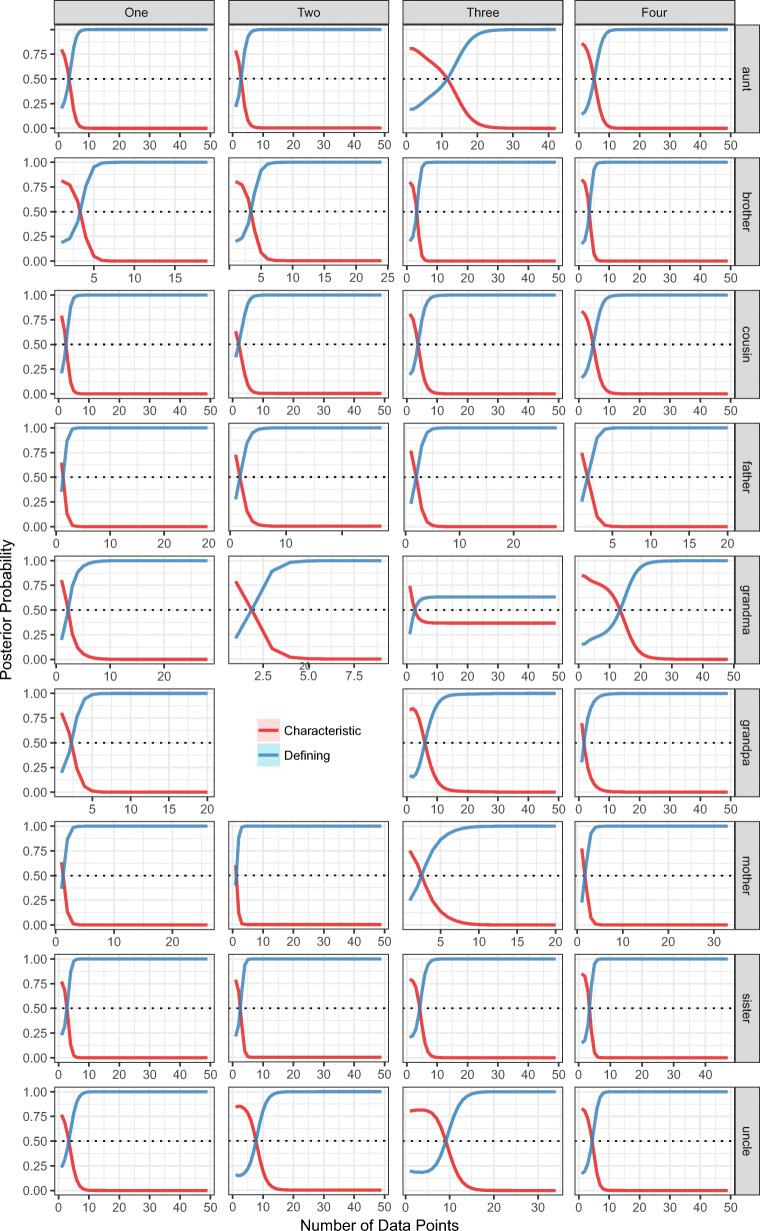
Average posterior probability of using a characteristic or a defining hypothesis (*y*-axis) as a function of the amount of data observed (*x*-axis) for words (*rows*) and informants (*columns*). *Shaded regions* reflect 95*%* bootstrapped confidence intervals. For all words, there is a characteristic-to-defining shift

Figure 7 plots the posterior probability of entertaining either a characteristic or defining hypothesis (*y*-axis) as a function of the amount of data observed (*x*-axis). For all of the words,^15^ we observe the characteristic-to-defining shift—i.e., the probability of entertaining a characteristic hypothesis is initially greater than the probability of entertaining a defining hypothesis. This means that a simple conceptual learning model shows a characteristic-to-defining shift purely due to the learning context—i.e., realistic data about logical relations and characteristic features. As these graphs average over the exact data points a learner observes, they hide the early preference for concrete referents; however, when plotted in terms of unique data points the early preference for concrete referents holds.

To further illustrate why the model exhibits this the characteristic-to-defining shift, we have replicated the table from Mollica, Wade, and Piantadosi (2017) as Table 4, which contains the three most likely hypotheses at different data amounts for Informant One’s simulated learning of grandma. Recall from the Model Outcomes that before seeing data, the model prefers simpler hypotheses that tend to over-extend. As the model sees more data points, the broad over-extensions narrows to better approximate the data. This is present in Table 4 as after seeing 3 data points, the extensions narrow from, for example, all women in the context to the outgoing individuals in the context, which include both of our informant’s grandmas as well as an aunt and a cousin. Importantly, the hypotheses that are favored after three data points are characteristic in nature yet imperfect in representing the concept. At one data point after the shift (i.e., the 13^*th*^ data point), the most likely hypothesis still over-extends (in Table 4 by including grandpas) and is defining in nature; however, there still is mass on characteristic hypotheses. As the model observes more data, the expected extensions will continue to narrow until the correct concept for grandma is the most probable.^16^

**Table 4:** Best hypotheses for Informant One learning grandma at three different time points

	Hypothesis	Posterior Probability
Before seeing data	X (i.e., the speaker)	0.354
	male(X)	0.006
	complement(X)	0.006
After seeing 3 data points	outgoing(Yes)	0.283
	nosy(Yes)	0.283
	small(Yes)	0.084
One data point after shift	parents(parents(X))	0.289
	female(parents(parents(X)))	0.268
	outgoing(Yes)	0.219

It is important to note that our model does not have a discrete change in processing or representation as appealed to by previous research (e.g., Kemler, 1983). Additionally, our model had access to abstraction from the outset of learning. Recall from Model Outcomes that without a bias promoting concrete referents, the model without characteristic features had a 25*%* chance of using abstraction after only observing a single data point (Fig. 3). Therefore, Piaget and Inhelder (1969)’s explanation, that the characteristic-to-defining shift reflects the *development* of abstraction, is not required. Our model shows that a rational learner would still undergo a characteristic-to-defining shift even if they had perfect access to the data and the ability to abstract from the outset of learning simply because characteristic features are simple and explain children’s initial data well. As children observe more data, children can justify more complex defining hypotheses if and when characteristic features fail to explain the data. If the characteristic features perfectly explain the data, children should never switch to defining hypotheses. Perhaps this is why the characteristic-to-defining shift is only observed in some conceptual domains and absent in others.

### Order of acquisition: Simplicity and data distributions

The extent to which simplicity, as opposed to experience, drives the order of acquisition of kinship terms is an open question. Previous research has found that American children tend to acquire kinship terms in a specific order: mother/father, brother/sister, grandpa/grandma, aunt/uncle and cousin. Haviland and Clark (1974) first explained this in terms of simplicity, measured as the number of predicates in first order logic required to define the kinship term. They later revised their account to additionally penalize reusing the same relational predicate (e.g., [X parent A][A parent Y] is more complicated than [X parent A][A child Y]). Other researchers have argued that data and the environment drive the order of kinship term acquisition. Benson and Anglin (1987) had parents rank order how frequently children spend time with, hear about or talk about twelve different kinship terms. They found that children’s experience with different kinship relations correlated with their observed order of acquisition. In our model, we can directly pit experience against simplicity and evaluate these theoretical hypotheses to determine if simplicity or experience drive the order of acquisition.

#### Implementation details

Here, we use the model to evaluate the predicted order of acquisition under several sets of assumptions. Starting with *simplicity*, our initial prior distribution over hypotheses (i.e., the PCFG in Table 1) mostly aligns with Haviland and Clark (1974)’s original formulation of simplicity, as seen in Table 5. If the likelihood of a data point across words was equal and data comes at a uniform rate for each word, we would expect to recover this order of acquisition. However, under the size-principle, the likelihood of a data point is not equal across words in this context, and CHILDES frequencies suggest that the frequency distribution for kinship terms is not uniform either^17^ (MacWhinney, 2000). Further, CHILDES frequency estimates differ from the surveys of Benson and Anglin (1987) and a larger corpus analysis of kinship term use across Indo-European languages (Rácz, Passmore, Sheard, & Jordan, 2019), which finds that frequency decreases as genealogical distance increases.^18^

**Table 5:** Complexity in terms of Haviland and Clark (1974) aligns with the prior probability of our model

Empirical Order	Word	Original H&C Order & Formalization	Log Prior	CHILDES Freq.
1	*mother*	Level I: [X parent Y][female]	-9.457	6812
1	*father*	Level I: [X parent Y][male]	-9.457	3605
2	*brother*	Level III: [X child A][A parent Y][male]	-13.146	41
2	*sister*	Level III: [X child A][A parent Y][female]	-13.146	89
3	*grandma*	Level II: [X parent A][A parent Y][female]	-13.146	526
3	*grandpa*	Level II: [X parent A][A parent Y][male]	-13.146	199
4	*aunt*	Level IV: [X sib A][A parent Y][female]	-19.320	97
4	*uncle*	Level IV: [X sib A][A parent Y][male]	-19.320	68
4	*cousin*	Level IV: [X child A][A sib B][B parent Y]	-18.627	14

Contrary to Benson and Anglin (1987)’s survey, CHILDES frequencies do not align with order of acquisition

Following Benson and Anglin (1987)’s surveys, we assume that children are more likely to be spoken to by people closer to them, and children are more likely to hear about people who are closer to them. We add these assumptions by sampling data from two Zifpian distributions over referents based on their distance to the speaker (see methods for details). In this, the relative grouping of different kinship relations will influence acquisition. For example, grandparents tend to be closer than cousins in our dataset, which would bias the learner to acquire grandparent before cousin. Second, the distance ranking of individuals that have the same kinship relationship will influence acquisition. For example, consider a learner with two uncles, one married-in and ranked more distant and one by-blood ranked closer. Due to distance on the tree, the learner will get data about the married-in uncle less frequently, which can delay their ability to acquire uncle because the learner must wait longer for data that teases apart the adult-like hypothesis from candidate hypotheses like *male*(*child*(*parents*(*parents*(*X*)))) that don’t capture uncles by marriage.

For our analysis, we factorially manipulated the model’s prior (Uniform/Simplicity) and the data distribution (Uniform/CHILDES/Zipfian). For each set of assumptions, we simulated 1000 data sets of 1000 data points from the tree in Fig. 1 and ran the learning model with only the base primitives to measure the probability that kinship terms are acquired in a specific order.^19^

**Fig. 8:**
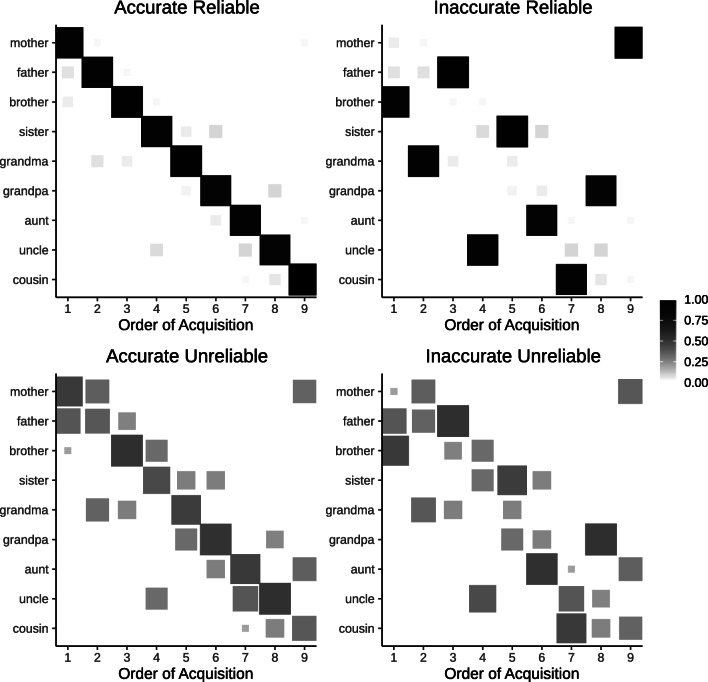
Possible patterns of order of acquisition. The *x*-axis reflects the ordinal position of acquisition. The *y*-axis represents each word. The tiles are filled according to the probability of acquisition. Words that have zero probability at a given ordinal position are omitted

Figure 8 illustrates four possible patterns that we might see with these simulations: an accurate and reliable order of acquisition (top left panel), an inaccurate, reliable order (top right), an accurate, unreliable order (bottom left) and an inaccurate, unreliable order (bottom right). In each panel, the *x*-axis reflects the ordinal position in which words were learned. The fill reflects the probability that a word was acquired at that time. If the order of acquisition is reliable, there should be only one probable word acquired at each ordinal position (top panels of Fig. 8). Whereas, if the order of acquisition is unreliable, there should be several probable words at each ordinal position (bottom panels of Fig. 8). We will quantitatively describe consistency using entropy (low entropy means more consistent) and describe the relationship between simulated orders and the attested order using Kendall’s tau correlation.^20^

#### Results and discussions

**Fig. 9:**
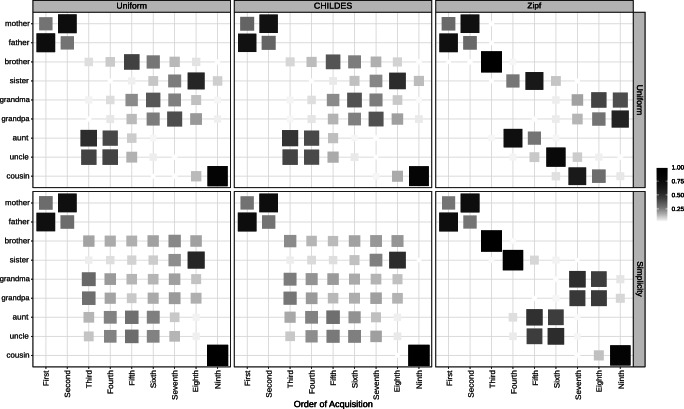
Simulations of the order of acquisition of kinship terms as a function of changes in environmental data distributions (*columns*) and the inductive biases of the learner (*rows*). A tiny amount of random noise was added to probabilities in each simulation to settle ties

Our simulations are plotted in Fig. 9 and quantitative descriptors are provided in Table 6. As a sanity check, we can see if the model would predict the empirical order of acquisition without simplicity or experience (top left corner). With a uniform prior and random input, the model does not closely match the attested order. Instead the likelihood imposes a relatively unreliable ordering favoring aunts and uncles. Comparison across the top row shows the influence of the assumed data distribution: matching frequencies to CHILDES results in little qualitative change, suggesting that the bias in word frequency distribution is not skewed enough to reliably alter the order of acquisition. Looking at the top right panel, using a Zipfian distribution increases the consistency of the predicted order of acquisition; however, the consistent trajectory does not closely follow attested order of acquisition (e.g., predicting *uncle* before *sister*). Taken together, experience alone does not seem sufficient to predict the empirical order.

**Table 6:** Quantitative description of consistency and correlation to attested order of acquisition

Prior	Environment	Joint Entropy	Rank Correlation
Simplicity	CHILDES	3.43	0.475 [0.197, 0.704]
Simplicity	Uniform	3.42	0.469 [0.254, 0.704]
Simplicity	Zipf	2.83	0.687 [0.592, 0.761]
Uniform	CHILDES	3.28	0.365 [0.197, 0.535]
Uniform	Uniform	3.25	0.365 [0.197, 0.535]
Uniform	Zipf	2.96	0.611 [0.479, 0.761]

Intervals reflect 95% posterior weighted interval. For reference, *τ* = 0.535 would be considered a significant correlation

Comparing the top and bottom left column, we can analyze the influence of the prior (Uniform vs. Simplicity). Using a simplicity prior pushes against the likelihood’s influence resulting in a less consistent pattern. Importantly, the less consistent pattern pushes off the incorrect ordering imposed by the likelihood. We can see the interaction between the simplicity prior and different data distributions in the bottom center and right panels. Looking at the center panel, the model predictions do not qualitatively change when adding a CHILDES frequency distribution. However, adding a Zipfian bias to a simplicity prior increases the consistency of the predictions (similar to the uniform prior case). Importantly, the predicted trajectory significantly correlates with attested trajectory, although imperfectly. This analysis suggests that the simplicity-based prior we have used throughout the paper has potential to explain detailed patterns of the timing of acquisition, although the predictions are dependent on the specific data distributions assumed. Both simplicity and experience drive the order of acquisition of kinship terms.

The discrepancies between empirical order of acquisition and our model predictions might be explained by how we assigned distances in the tree. For example, if aunt/uncles were further from the learner than grandparents, we might expect grandparents to be acquired earlier. Ideally, these distances would be informed by cultural/environmental factors that further constrain the learning problem. For example, we would expect matrilinear/patrilineal residence patterns to influence the order of acquisition through these distances. In our simulations, differences between concepts of the same complexity (e.g., grandma and grandpa) are slightly influenced by ties such that the alphabetical order appears dominant in Fig. 9 where there is likely no bias. Importantly, under this Zipfian environmental distribution the model still shows under-extension, over-generalization and the characteristic-to-defining shift (Mollica, Wade, & Piantadosi, 2017).

## General discussion

By framing kinship concept induction as logical program induction, we have demonstrated how simplicity and the size principle predict several of the empirical phenomena seen in children’s acquisition. Specifically, an ideal learner model incorporating these principles learns the kinship system consistent with its input, offering a cross-linguistic proof of learnability that works for typologically diverse kinship systems. The trade-off between simplicity and the size principle drives the model to predict both an early preference for concrete reference and patterns of over-generalization broadly consistent with the patterns in children’s behavior, including the characteristic-to-defining shift. Additionally, our model provides a novel explanation for the characteristic-to-defining shift seen in children’s early understanding of words, highlighting the role of the learning context instead of proposing discrete changes in representation and processing. Lastly, the model has addressed open theoretical questions about the forces driving the order of acquisition of kinship terms in English.

Beyond kinship, our model derives strong predictions for how conceptual development should unfold over time from first principles—i.e., simplicity and strong sampling. Previous research has highlighted the limitations of using children’s early word use as evidence for their comprehension, arguing that performance limitations and pragmatic reasoning heavily influences early productions (Fremgen & Fay, 1980; Bloom, 1973). Having independent predictions for how conceptual knowledge unfolds over time provides leverage to further investigate how conceptual knowledge interacts with developmental models of retrieval and pragmatic reasoning. For example, can out-of-vocabulary over-extensions be explained better by under-developed concepts or pragmatic reasoning with adult-like semantics for a limited number of words (Xu & Pinto, in press)?

Table 7 outlines each behavioral phenomena this model explains and the components of the model that do so. There are two ways in which the behavioral predictions of our computational model can be used. First, experiments can be designed to directly assess components of the model, and the learning environment. For example, we can evaluate the model predictions under different primitive functions against children’s patterns of generalization as in the tradition of componential analysis. Similarly, assumptions about how children use data (i.e., the likelihood function) and the inductive biases they bring to the learning task make different predictions for patterns of generalization and the timing of those behaviors. The model also makes predictions for if and when a learning context should result in a characteristic-to-defining shift. Second, this model can be used as a baseline or normative model for comparison against other theories of conceptual learning and for the development of theories of related processes. Take for example a foundational debate in anthropology that kinship is uniquely disposed to address (Kroeber, 1909; Rivers, 1914): do we learn the structures in the world or do we learn the conventions of lexical production through linguistic structure? Our model shows how a learner should behave if their goal was learning the structure in the world. Comparing the predictions of our model with those of formal models built to learn from linguistic structure would give us leverage to tell when and to what extent children are learning from world structure or through linguistic structure. Additionally, the model makes predictions of how children’s competence should change as a function of data, which has the potential to aid the construction of theoretical models of word use and early learning.

**Table 7:** Summary of the empirical behavior, how the model explains this behavior and the behavioral predictions to be generated by the model

Empirical behavior	Model explanation	Behavioral predictions
Cross-linguistic learnability	Inductive learning	The number of data points before acquisition
Under-extension	Local data distribution	The number of data points before abstraction.
Over-generalization	Trade-off between prior and likelihood	The pattern of generalization at each data amount
Characteristic-to-defining shift	Learning context	The presence of and the number of data points before the shift
Order of Acquisition	Environmental experience	The order of acquisition and number of data points before each term
		is acquired

With regards to kinship specifically, the model contributes to the long-standing challenge of identifying constraints on possible kinship systems. For over a century, anthropologists and linguists have attempted to explain why there is such rich but constrained diversity in kinship systems and how this diversity and structure has arisen. Traditionally studies have approached this problem viewing kinship systems as a mathematical object (e.g., Goodenough, 1956; Read, 2007), with little concern for the psychological reality. In the last decade, formal modelling has started incorporating functional pressures to explain kinship (Jones, 2010; Kemp & Regier, 2012). While these endeavors have some success explaining why there is diversity in kinship systems, they fail to explain the rich structure within kinship diversity (Passmore et al., 2021) and how kinship systems have evolved (Passmore & Jordan, 2020). In order to understand evolutionary trajectories, we will need to combine constraints inspired by mathematical descriptions of kinship structure (e.g., Jones, 2010) with constraints inspired by viewing kinship as a cognitive technology, including acquisition. The model can serve as an additional constraint to explain why there is repeated structure across attested kinship systems despite the lack of support for universal models of evolutionary change (Passmore & Jordan, 2020).

An important direction for future models is to learn all of the parallel structures supporting kinship—i.e., how kinship terms (sometimes simultaneously) map to address, sociological and attitudinal structures. For example, it’s easy to imagine a child construing *uncle* in *Uncle Ben* as a term of address like *doctor* in *Doctor Octavius*. Similarly, kin terms can be used to express an attitude toward an individual. For example, calling an individual a *grandpa* because they go to sleep and wake up early. Furthermore, future work should seek to evaluate these systems against social reasoning behavior in addition to establishing reference. Future implementations of models in our framework could map kinship terms to different structure or simultaneously learning multiple mappings. Of course, these endeavors will also require a substantial investment in both experimental and observational data collection for kinship across cultures.

## Conclusion

Programs are a powerful representational scheme to formalize concepts because they have the ability to capture logical structure, features, and potentially graded or stochastic aspects of conceptual structure. A critical component of our program representation scheme is that our programs are functions of contexts. Concept deployment and language use are heavily context-sensitive and to generalize across contexts, thinkers need something like a program, which can operate over a given context. When combined with data-driven inductive approaches, programs not only capture the end state representation of concepts but provide rich behavioral predictions across the entire developmental trajectory, including phenomena like the characteristic-to-defining shift in a single model.

## Methods

### Generating the hypothesis space

To construct a finite lexicon space appropriate for our analyses, we utilized a variety of Markov Chain Monte-Carlo methods to draw samples from the posterior distribution over lexicons at different data amounts. Our model is implemented using LOTLib, a Language of Thought library for python (Piantadosi, 2014a). Here a lexicon is a collection of hypotheses, one per kinship term. First, we searched the space of all possible lexicons using MCMC, resulting in many partially correct lexicons. Across all of these lexicons, every word was learned and therefore, the learning trajectory for each word was present in the space. Nonetheless, few if any lexicons contained the correct hypothesis for all of the words, which is important to ensure that the finite approximation of the space that we use contains as many lexicons that are developmentally plausible as possible. In our second phase, we mixed the hypotheses generated in the first phase to construct lexicons that contained the developmental trajectories of multiple words. A small percentage of these lexicons contained correct hypotheses for all of the words. Phase one and two combined generated too many lexicons to tractably analyze further. Therefore, we truncated the space by normalizing the lexicons and selecting the top 1000 hypotheses at various data amounts. For our main analyses, we collapse across lexicons and analyze developmental trajectories for each word independently to avoid any complications with not having a complete lexicon space.

To generate an initial set of hypotheses, we used the Metropolis-Hastings algorithm using tree-regeneration proposals following (Goodman et al., 2008; Piantadosi et al., 2012). For each language, we ran 16 chains at each of 25 equally spaced data amounts between 10 and 250. Due to memory limitations, we only saved the top 100 best lexicons from each chain. For English, Pukapukan, and Yanomaman lexicons, each chain was run for 1 million steps. For Turkish, we first ran five chains for 3 million steps on a smaller lexicon—i.e., the search did not include the three words for grandparents or the word for cousin. We then ran five chains for 3 million steps on the full lexicon. Few if any lexicons resulting from this search contained the correct hypothesis for all words; however, across all lexicons the correct hypothesis for every word was learned.

In our second phase, we used Gibbs sampling to mix the hypotheses generated in the first phase, constructing lexicons that contained the developmental trajectories of multiple words. A small percentage of these lexicons contained correct hypotheses for all of the words. Phase one and two combined generated too many lexicons to tractably analyze further (around 200,000 nine-word lexicons for English). Therefore, we truncated the space by normalizing the likelihoods and selecting the top 1000 lexicons at various data amounts favoring lower amounts (eight equally spaced intervals between 1 and 25, and six equal intervals between 25 and 250 data points). For the analyses presented in the main text, we marginalize over lexicons to analyze hypotheses for different kinship terms independently. As hypotheses are included in the space based on their performance at varying data amounts, we normalize the likelihood by simulating 1000 data points, computing the likelihood of each hypothesis and taking the average likelihood for each hypothesis.

### Learnability, *F*_1_ and over-extension analyses

To evaluate if a hypothesis $\hat {h}$ was correct, we compared the hypothesis’s extension to the hand-constructed, ground truth hypothesis *h* for each kinship term system. We obtain the trajectories for posterior weighted accuracy, precision and recall by marginalizing over hypotheses at each data amount. For example, the posterior weighted accuracy is given by:

4 $$  P(\hat{h}=h|d) = {\sum}^{\mathcal{H}} \delta_{\hat{h}h} P(h|d). $$

We adopt this same approach to estimate the extension probability for each referent *x* in a context as a function of data:

5 $$ P(x|d) = {\sum}^{\mathcal{H}} P(x \in |h|) P(h|d), $$

where *P*(*x* ∈|*h*|) is given by:

6 $$ P(x \in |h|) = \begin{cases} 1 \text{ if } x \in |h|\\ 0 \text{ else.} \end{cases} $$

### Concrete reference analysis

As concrete reference is heavily influenced by local data distributions, we constructed a fixed data set of five unique data points for uncle and ran one MCMC chain 100,000 steps for each amount of data. We collected the top 100 hypotheses from each chain to use for analysis. We operationalize abstraction as the probability the hypothesis is a function of the speaker:

7 $$ P(r_{SET \rightarrow p} \in h) = \begin{cases} 1 \text{ if } r_{SET \rightarrow p} \in h\\ 0 \text{ else} \end{cases}. $$

The posterior probability of using abstraction at a given data amount is therefore:

8 $$ P(r_{SET \rightarrow p}|d) = {\sum}^{\mathcal{H}} P(r_{SET \rightarrow p} \in h) P(h|d). $$

We manipulate the prior bias for concrete reference by changing the PCFG production probabilities given in Table 1, which influences the prior probability following Equation 2.

### Characteristic-to-defining shift

We build the hypothesis space for characteristic and defining features separately for each informant. To gather defining hypotheses, we ran seven chains at each of 25 equally spaced data amounts between 10 and 250 using the PCFG in Table 1 for 500,000 steps. To gather characteristic hypotheses, we ran seven chains at each of 25 equally spaced data amounts between 10 and 250 using the PCFG in Table 3 for 500,000 steps. Due to memory limitations, we only saved the top 100 best lexicons from each chain. For each informant, the defining and characteristic hypotheses were concatenated to form a single finite hypothesis space. As our analyses collapsed over lexicons, we did not perform Gibbs sampling as above.

We replicate the learnability and *F*_1_ analyses (described in Appendix B) using the same methods described above. Our analysis of the characteristic-to-defining shift is similar to our analysis of concrete referents. The posterior probability of using a characteristic hypothesis at a given data amount is

9 $$ P(r_{FSET \rightarrow \text{feature}}|d) = {\sum}^{\mathcal{H}} P(r_{FSET \rightarrow \text{feature}} \in h) P(h|d), $$

where $P(r_{FSET \rightarrow \text {feature}} \in h)$ is:

10 $$ P(r_{FSET \rightarrow \text{feature}} \in h) = \begin{cases} 1 \text{ if } r_{FSET \rightarrow \text{feature}} \in h\\ 0 \text{ else.} \end{cases} $$

### Order of acquisition analysis

For the uniform data distribution, we sampled 1000 different datasets each containing 1000 data points from a uniform distribution over all possible true data points. For the CHILDES data distribution, we sampled 1000 different datasets each containing 1000 data points as follows. A kinship term *w* is sampled from a multinomial distribution with *𝜃* values reflecting CHILDES frequencies. Given that term, a speaker-referent pair (*x*,*p*) is sampled uniformly from all possible speaker-referent pairs.

11 $$ w \sim \text{Multinomial}(\theta) $$

12 $$ (x,p) \sim \text{Uniform}(\{(x,p)\}) $$

To simulate *experience* according to Benson and Anglin (1987), we modified the data generating process. For each data point, speakers ranked closer in distance to the learner are more likely to be sampled than data from speakers ranked distant to the learner. Conditioned on a speaker and a word, valid referents ranked closer to the learner are more likely to be sampled than referents ranked distant to the learner. We implement both of these models with the same noise model used in Equation 3. First, a kinship term is sampled following (11). Conditioned on a word, a speaker is sampled from a Zipfian distribution over the set of all possible speakers of that word $\mathcal {X}_{w}$:

13 $$  P(x|w) \sim \frac{\alpha d_{x}^{-s}}{{\sum}_{x \in \mathcal{X}_{w}} d_{x}^{-s}} + \frac{(1-\alpha)}{|X|} , $$

where *d*_*x*_ is the ranking of the speaker *x*, *s* is the Zipfian exponent and *X* is the set of all individuals in the tree context. Conditioned on the word and speaker, a referent is sampled from a Zipfian distribution over the set of all possible referents for that word and speaker $\mathcal {P}_{wx}$:

14 $$  P(p|x,w) \sim \frac{\alpha d_{p}^{-s}}{{\sum}_{p \in \mathcal{P}_{wx}} d_{p}^{-s}} + \frac{(1-\alpha)}{|X|} . $$

For our analyses, *s* = 1, reflecting the typical bias observed in texts (Piantadosi, 2014b). We assigned distances to the tree context in Fig. 1 by fixing the learner as the central female in the youngest generation that had both a brother and a sister, and assigning relatives closer in Euclidean distance smaller distance values. The assignment of distance in our informant provided data suggests this relationship has great individual variability, so we refrain from making strong predictions about the order of acquisition for individual terms. For the Zipfian distribution, we sampled 1000 different datasets each containing 1000 data points as outlined in Equations 13 and 14.

For all simulations schemes, we calculate the posterior accuracy of each hypothesis as a function of data following Equation 4 after each data point is sampled. If the posterior weighted accuracy is greater than or equal to 0.99, we mark the word as learned and record its ordinal position. Ties were resolved alphabetically. As a result, we do not make strong predictions about order of acquisition for equally complex concepts (e.g., the relative ordering of mother and father), which often pattern alphabetically in our simulations.

## Figures and Tables

**Fig. 10:**
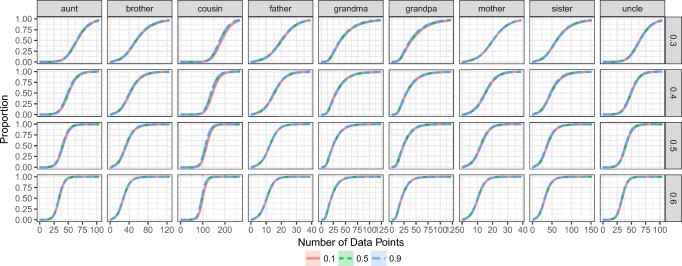
Posterior weighted accuracy (*y*-axis) as a function of data (*x*-axis) for models with different sampling assumptions (linetype and color) for different words (*columns*) and environmental reliability values (*rows*). The virtually invisible shaded regions reflect 3 standard errors of the mean

**Fig. 11:**
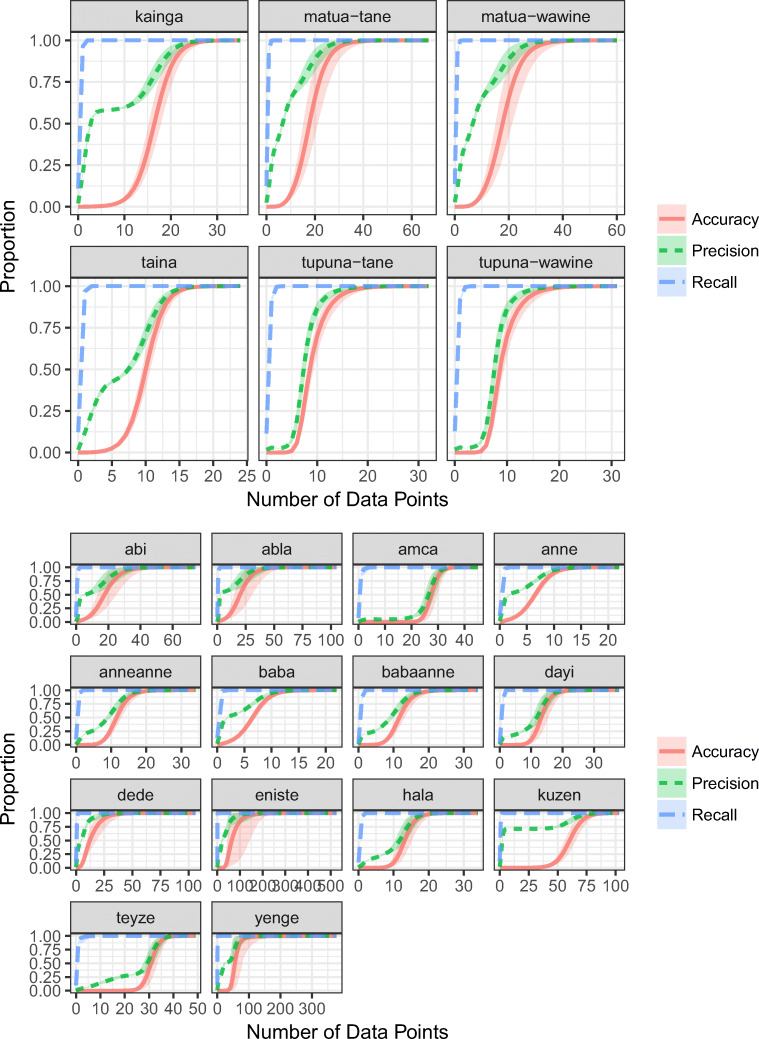

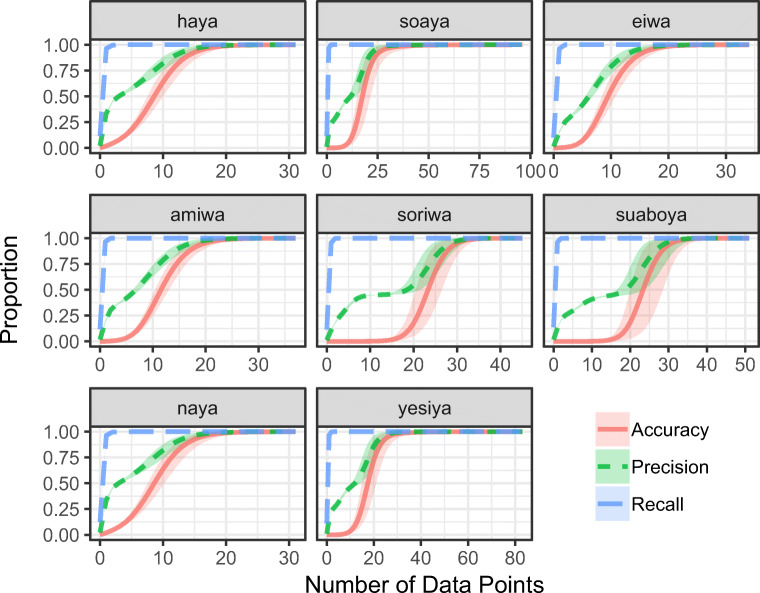
Average lexicon posterior-weighted accuracy, precision and recall for each word as a function of data points. Precision greater than recall is a hallmark of over-generalization. *Shaded regions* represent 95*%* bootstrapped confidence intervals

**Fig. 12:**
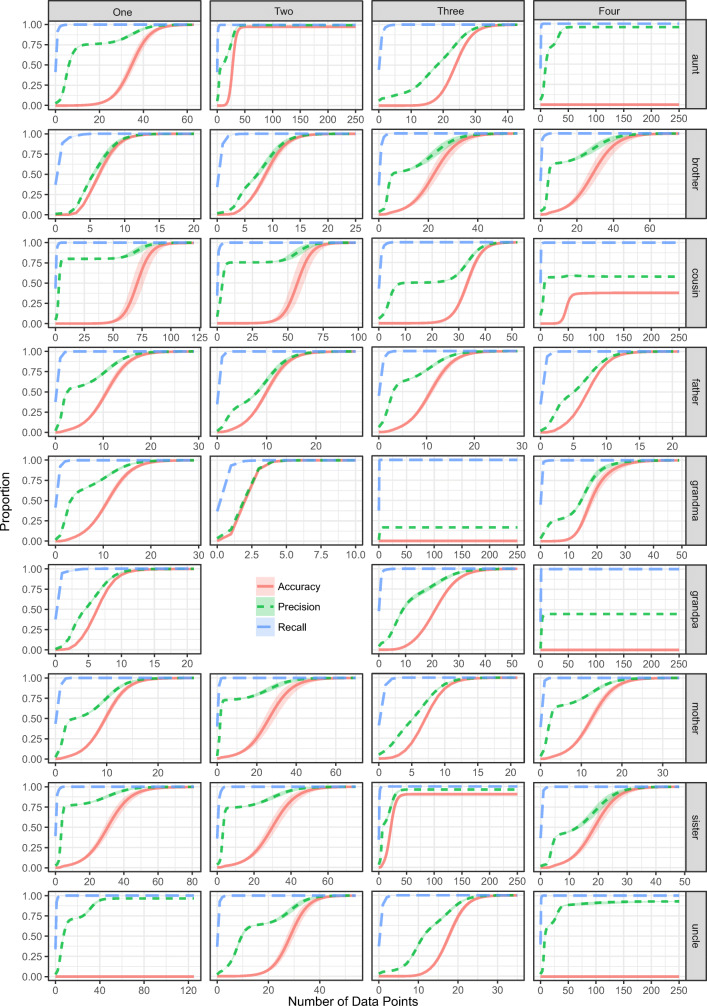
Average lexicon posterior-weighted accuracy, precision and recall for each word as a function of data points. Precision greater than recall is a hallmark of over-generalization. *Shaded regions* represent 95*%* bootstrapped confidence intervals

**Fig. 13:**
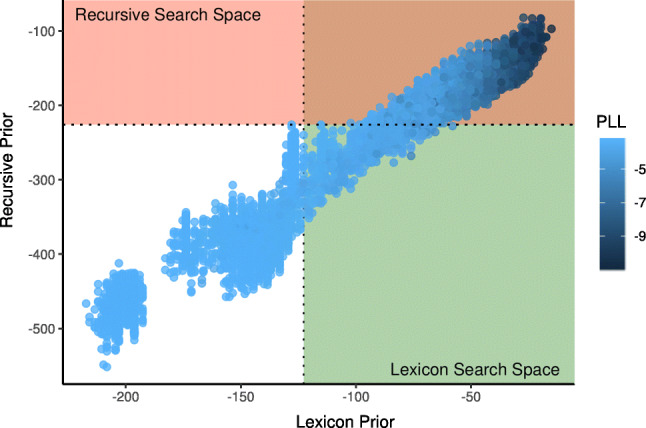
The English lexicons are plotted as a function of the recursive (compression) and lexicon prior. The color of each point represents the point log likelihood (PLL) of the lexicon. If the learner searched the space starting from the simplest to the most complex lexicon and terminated at the first correct lexicon, they would have to search a smaller space under a compression prior (*red shade*) than under a lexicon prior (*green shade*). Importantly, the developmental trajectory is not predicted under the recursive prior without additional assumptions about the complexity/development of recursion

## Data Availability

The code is available at https://github.com/MollicaF/LogicalWordLearninghttps://github.com/MollicaF/ https://github.com/MollicaF/LogicalWordLearningLogicalWordLearning.

## Appendix A: Alpha analysis

Navarro et al., (2012) investigated how the reliability parameter *α*, which mixes between strong and weak sampling influences an inductive generalization task. They simulated environments where the data was generated to be reliable 30–60% of the time, and checked how distinguishable a noisy size-principle likelihood with varying reliability parameter *α* would be from pure strong sampling (*α* = 1). They found that in the limit of data, models with reliability parameters as low as 0.1 converge to the predictions of strong sampling. We parametrically vary the reliability of the environment by simulating data with 30 − 60*%* reliability and set our model’s reliability parameter to either 0.1, 0.5, and 0.9 to gauge whether learning in our simulations will be robust to unreliable environments and different reliability assumptions. As can be seen in Fig. 10, we find no qualitative differences in learning across reliability assumptions and environments.

## Appendix B: *F*_1_ score plots

As described in the main text, *F*_1_ score plots are a visualization of learnability and over-generalization. Each figure in this appendix plots the posterior weighted accuracy, precision and recall (*y*-axis) as a function of data (*x*-axis). Accuracy reflects the probability that the model has acquired the adult-like concept for that kinship term. Recall corresponds to the probability that the model will recognize a correct referent, and is given by:

15 $$ \frac{{\sum}_{x \in \hat{h}} [x \in h] }{|h|}, $$

where *x* is a referent, $\hat {h}$ is the proposed hypothesis, *h* is the ground truth hypothesis. Precision corresponds to the probability that the model will propose a correct referent, and is given by:

16 $$ \frac{{\sum}_{x \in \hat{h}} [x \in h] }{|\hat{h}|}. $$

When recall is greater than precision, the model is over-extending the term.

Figure 11 displays the *F*_1_ plots for Pukapuka, Turkish and Yanomamö. As shown in the main text, the model learns the correct extension for every word. As expected, the posterior weighted recall is greater than the posterior weighted precision for every word, suggesting that the model over-extends the meaning of kinship terms. Predictions for the pattern of over-extension for each word is provided in supplemental material.

### B.1 The characteristic-to-defining shift

Figure 13 displays the *F*_1_ plots for each of our informants. For all words, posterior weighted recall is greater than posterior weighted precision, consistent with over-extension of kinship words. As discussed in the main text, the model fails to learn the correct hypothesis for some words due to the impoverished input/context. That being said, the model always learns a hypothesis that is consistent with its input. If we had provided evidence from multiple family tree contexts, we expect the model to learn the adult-like extension for all of the concepts. This suggests that having evidence from multiple families is likely an important property of the kinship data that children use to learn their kinship terms.

In the majority of cases where the model does not acquire the correct extension, the conventional hypothesis was blocked by a hypothesis that overfit the context. For example, Informant 3 overfits for grandma and Informant 4 overfits for grandpa because there is only one of those relations in their family tree. Hence, it is sufficient to just point to that person. Informant 2 does not learn aunt, Informant 3 does not learn sister and Informant 4 does not learn cousin for similar reasons. In these cases, the conventional hypotheses do have some posterior probability (as evidenced in Fig. 13 by non-zero Accuracy) but do not come to dominate the posterior distribution of possible hypotheses. The conventional hypotheses are blocked by hypotheses that are less complex, explain the observed data, but would not generalize properly across contexts.

Instead of overfitting, Informant 1 and 4 do not learn the conventional hypotheses for aunt and uncle because there are children out of wedlock, which complicates how we have defined the conventional hypotheses. Importantly, the maximum-a-posteriori, or best, hypothesis recovered by the model actually generalizes correctly over trees without out of wedlock children. Informant 2 does not have any grandfathers in their family tree context and, therefore, the model never receives data to learn grandpa.

## Appendix C: Learning an inter-related system

Throughout the paper, we have described a model that learns kinship terms independently of each other. One trivial way to implement learning an inter-related system would be to change the likelihood function to operate over the lexicon instead of individual words (e.g., Mollica, 2019). However, the more natural way to think of learning an inter-related system like kinship would be to allow for recursive calls.^21^ For example, a learner might use their current concept for brother in their concept for uncle. We have implemented recursive calls in the model; however, despite multiple attempts, we were unable to construct an acceptable lexicon space to evaluate the model against developmental behavior. Without a proper finite approximation to the space of probable lexicons, there are no guarantees that any “conclusions” drawn will be robust.

One common issue with the search was finding lexicons that only learned a subset of the words after a lengthy search process. In the main text, we could easily mix lexicons using Gibbs sampling to help ensure the relevant lexicons—i.e., lexicons that contain all high probability combinations of hypotheses across the developmental trajectory, were in our finite approximation of the space. Unfortunately, recursive calls introduces dependencies between words in a lexicon, which prohibits techniques like Gibbs sampling that rely on independence.

**Table 8:** An example local max lexicon when permitting recursive calls in the lexicon space

father	male(parent(X))
mother	female(parent(X))
brother	child(parent(X))
sister	female(brother(X))
uncle	male(brother(parent(X)))
aunt	female(brother(parent(X)))
cousin	difference(generation0(X), brother(X))

Another common issue was the presence of local maxima in search (illustrated in 8. Often the model would construct a useful primitive (e.g., *sibling*) instead of the definition of a word (e.g., *brother*), which blocks that word from being acquired. In the example lexicon in Table 8, sister, uncle, aunt and cousin are all defined in terms of the learner’s hypothesis for brother. The learner’s hypothesis for brother is incorrect and would be better glossed as *sibling*. The problem with this local maxima is that any change to brother to fix it would result in errors for sister, uncle, aunt and cousin. Therefore, the sampling chain cannot propose a better lexicon and is essentially stuck.

Due to the search issues, we adopted a different tactic to explore the effect of recursion on kinship learning. Hypotheses with recursive calls have extensionally-equivalent hypotheses defined in terms of the base primitives. For example from Table 8, sister could be expressed as female(child(parent(X))). Being extensionally equivalent, the two hypotheses have the same likelihood. The only difference on the posterior probability is in the prior. Recursive hypotheses should be simpler and thus more probable. Therefore, we can change the prior distribution over our existing hypothesis space to behave equivalently as if it was recursive. We capture the same intuitions as recursion using the Lempel-Ziv compression of the lexicon in terms of the grammar as a prior over lexicons. This prior distribution favors the reuse of specific combinations of primitives in the lexicon similar to the recursive calls in Table 8.

We found that when using a compression prior, the model predicts an inductive leap from most of the kinship terms not being properly acquired to all of the kinship terms being learned. We see this leap because the correct lexicon under the compression prior is significantly less complex than the lexicons required in search space to get you there (Fig. 13). To remove this inductive leap, we could add a parameter that penalizes recursion (Piantadosi et al., 2012); however, we think that the better explanation would be through the development and integration of a more cognitively grounded notion of hypothesis generation—i.e., an algorithmic level explanation.

## Appendix D: Mutual exclusivity through pragmatics

At first glance, our model fails to capture *mutual exclusivity*, or the bias for a referent to map to a single word (Golinkoff, Hirsh-Pasek, Bailey, & Wenger, 1992; Markman & Wachtel, 1988). Our model often predicts patterns of over-extension where a term like *aunt* would include a referent that overlaps with another term like the speaker’s mother. This is counter-intuitive and anecdotally it’s rare that children would actually use the word *aunt* to refer to their mother. There are several ways we could add mutual exclusivity directly to the model. For example, (Markman & Wachtel, 1988; Markman, Wasow, & Hansen, 2003) suggested that children have an inductive bias specifically for mutual exclusivity. Instead, we suggest that mutual exclusivity should be handled by the natural pragmatic reasoning of a communication task. Let’s take for example, the pragmatic reasoning model proposed by (Frank & Goodman, 2012). We will consider a learner sitting in a room with their aunt and mother with the kinship concepts in Table 9.

**Table 9:** Example leaner

Concept	Hypothesis	Relative Frequency
aunt	female(generation1(X))	0.3
mother	female(parent(X))	0.7

First, let’s look at production. If our learner is a pragmatic speaker and needs to refer to a target referent *r* in the context *C*, they should select the word *w* in their vocabulary that is most likely to extend to the target referent *P*(*w*|*r*,*C*). This can be formalized using Bayes rule as:

17 $$ P(w|r, C) = \frac{P(r|w, C) P(w)}{{\sum}_{w} P(r|w, C) P(w)} , $$

where P(r|w, C) is our noisy size-principle likelihood Equation 3. Using this equation, if the learner needs to refer to their mother, they should use *mother* because there is an 82*%* chance that *mother* would be used to refer to their target referent and only 12*%* chance that *aunt* would. If they need to refer to their aunt, they should use *aunt* because there is an 81*%* chance that *aunt* would be used to refer to their target and a 19*%* chance that *mother* does.

Now looking at comprehension. If our learner is a pragmatic listener, they will infer reference based on what a pragmatic speaker should do. Formally, the probability of a referent given a word is:

18 $$ P(r|w, C) \propto P(w|r, C) P(r|C). $$

Thus, the pragmatic listener propagates the mutual exclusivity bias of the pragmatic speaker. Assuming an equal prior on referents in the context, a pragmatic listener should understand *mom* to refer to their mother, as there is an 81*%* chance that *mom* refers to their mother, and *aunt* to refer to their aunt, as there is an 82*%* chance that *aunt* refers to their aunt.

Allowing this kind of pragmatic generalization is potentially beneficial for communicating out-of-vocabulary referents (Xu & Pinto, in press) and establishing reference with an ambiguous linguistic signal. To be clear though, we offer this explanation at the computation level. We are not claiming that children solve this pragmatic reasoning problem explicitly; however, they must implicitly solve this problem for successful and efficient communication regardless of their semantics. Our conclusion is just that we might be able to get mutual exclusivity from pragmatics without requiring an inductive bias. For further evidence against the inclusion of a specific inductive bias for mutual exclusivity, see Frank, Goodman, and Tenenbaum (2009), which discusses how a simplicity bias is sufficient to predict mutual exclusivity in the word-referent mapping problem. Similar observations can be drawn from several implemented models of cross-situational word learning (Fazly, Alishahi, & Stevenson, 2010; Kachergis, Yu, & Shiffrin, 2012; McMurray, Horst, & Samuelson, 2012). For a recent review and meta-analysis of mutual exclusivity, see Lewis, Cristiano, Lake, Kwan, and Frank (2020).

## Footnotes

1 Kinship as a construct potentially operates over multiple structures, including systems of address, sociological systems and social categories (Read, 2001, 2007). As a point of scope, we focus here on genealogical notions of kinship terms—i.e., kinship terms defined over a family tree.

2 Of course, the modelers are aware of the behavioral patterns, which is why we take care in laying out the model assumptions, where predictions are mutable and where further grounding is needed.

3 In the main text, we focus on learning independent kinship terms. A thorough analysis of learning inter-related system is beyond our current scope; however, we discuss our preliminary exploration of inter-related learning schemes in Appendix C.

4 Although see Mollica and Piantadosi (2015) for a first-order logic implementation of our model.

5 Our use of “primitive” reflects the atomic nature of the functions within the kinship domain and is not a claim about innateness.

6 Unlike linguistic or componential analyses, we do not intend for these base functions to be a complete account of all of the functions required for learning kinship systems or all of the functions children might bring to the task. For example, children would require primitives to compute relative age or patrilineage to learn some kinship systems (e.g., Japanese and Korean). It is easy to see how one could decompose certain primitives into one level less of abstraction (e.g., generation might be represented in terms of primitives that check for perceptual features) or how one could choose to augment a set at a greater level of abstraction (e.g., adding a sibling primitive). For any model of learning, the granularity and span of a hypothesis space depends on the characterization of the learning problem (Perfors, 2012). Our general findings will not strongly depend on any particular base function inventory; however, inventories can make different predictions about the precise pattern and timing of children’s behavior over learning.

7 Specifically, Chapais (2014) argue that we are innately predisposed to recognize maternal bonds and maternal siblings; however, paternal recognition is a derived human adaptation.

8 We make the assumption that programs do not return the speaker as referent—i.e., a bias against computing a kinship term as self-referential. For example, when a male speaker computes the set of his brothers *m**a**l**e*(*c**h**i**l**d**r**e**n*(*p**a**r**e**n**t*(*X*))) he excludes himself from the output.

9 In Supplementary Fig. 10, we emulate the simulations conducted by Navarro, Dry, and Lee (2012) to demonstrate that our main findings are robust under realistic values of *α*.

10 For ease of computational search, we modified two primitives used to capture the relations in Yanomamö compared to the other kinship systems. Specifically, we exclude “in-laws” when calculating generation for Yanomamö but include them for the other cultures. Second, we added a primitive that takes an individual as input and returns the set of adults who co-reside with the input individual only for Yanomamö. Our co-residence primitive is motivated by the patrilocal residence patterns and prescriptive cross-cousin marriage in Yanomamö society. Following patrilocality, a father’s brothers live locally and following strict cross-cousin marriage, a mother’s sisters are likely to live locally to her. Our modifications for Yanomamö was motivated primarily by a desire to decrease computational search time. That being said, related-generation and co-residing adults are plausibly noticed by children and would serve as a strong cue for relevant genealogical relationships in some kinship systems.

11 The specific patterns of over-generalization will depend heavily on the base functions and more empirical data is needed to distinguish between base function inventories.

12 Appendix B contains *F*_1_ score plots for every language and context simulated in this paper.

13 All family trees, feature matrices and code can be found at https://github.com/MollicaF/LogicalWordLearning.

14 The model could accommodate for this limitation by sampling across multiple contexts; however, this is computationally expensive to do for each of our informants. For computational efficiency, we only sample data for each informant within their context.

15 Informant 2 has no grandfather relations in their family tree context.

16 While we focus here on learning a single rule for kinship terms, it is more likely that adults retain several rules which they can flexibly deploy to determine kin relationships. For example, characteristic features of kin relations still influence human reasoning well after rule-like definitions have been learned (Lupyan, 2013).

17 As a larger point, it is not clear that children make use of every instance of a word in their environment as input for learning. While frequency is a good predictor of mean age of acquisition (Braginsky, Yurovsky, Marchman, & Frank, 2019), Mollica and Piantadosi (2017) inferred the rate of input and amount of input children require for learning and found that frequency was not strongly correlated with how many effective learning instances children required to learn a word. However, frequency was moderately correlated to the rate of effective learning instances children received.

18 Although, Rácz, Passmore, Sheard, and Jordan (2019) did not include grandparents in their analysis.

19 We returned to the simulated tree for practical convenience and because the sparseness of the solicited trees lead to incomplete learning of kinship terms (see Appendix B).

20 Statistics presented resolve the ties in the attested order alphabetically, which is consistent with how we handle ties in model predictions.
